# Fundus image analysis of retinitis pigmentosa using artificial intelligence

**DOI:** 10.1371/journal.pone.0354452

**Published:** 2026-07-24

**Authors:** Saki Ubukata, Kanato Masayoshi, Yusaku Katada, Lizhu Yang, Nobuhiro Ozawa, Mari Ibuki, Kazuno Negishi, Toshihide Kurihara

**Affiliations:** 1 Laboratory of Photobiology, Keio University School of Medicine, Tokyo, Japan; 2 Department of Ophthalmology, Keio University School of Medicine, Tokyo, Japan; Tehran University of Medical Sciences, IRAN, ISLAMIC REPUBLIC OF

## Abstract

Retinitis pigmentosa (RP) is a group of inherited retinal diseases that are caused by genetic defects that lead to progressive photoreceptor loss and eventual blindness. Early diagnosis would be helpful for effective management of the disease; however, many patients stay unaware of early symptoms. Meanwhile, fundus images are widely obtained during routine medical checkups but are underused for detecting RP. This study explores the effectiveness of finetuning deep learning models, pre-trained for general visual tasks, to identify RP from color fundus images. The dataset comprised 321 color fundus images from 201 Japanese subjects at Keio University Hospital, including 200 images from 107 patients with retinitis pigmentosa and 121 images from 94 non-retinitis pigmentosa subjects. Multiple images were available for some subjects. Using transfer learning, pretrained convolutional neural network models -VGG16, Resnet50, and InceptionV3- were finetuned to detect RP. As a result, Inception V3 achieved the best accuracy of 96.97%, which matches the average diagnostic accuracy of ophthalmologists. Gradient-weighted Class Activation Mapping (Grad-CAM) suggested that the model attended to clinically relevant fundus regions, including the peripheral retina and posterior pole, which may reflect features such as peripheral degenerative changes and retinal vascular attenuation. These findings support the potential interpretability of the finetuned model and suggest that deep learning may assist ophthalmologists in RP screening as a supportive tool.

## Introduction

Retinitis pigmentosa (RP) is an intractable and serious eye disease that affects approximately 2 million people worldwide [1]. RP is caused by degeneration of photoreceptors in the retina, resulting in the appearance of abnormal findings on fundus images, mainly pigmentation, in the periphery. As the pigmentation progresses, visual field testing and fundus imaging are performed to monitor the progress of the disease. Although there is no effective treatment for RP at this moment, research and development are actively underway [2,3]. Thus, it is important to diagnose and predict RP to smoothly guide potential future treatment. Early diagnosis also has the potential to reduce psychological burden by allowing more time to prepare for visual impairment in the future. However, detecting RP in its early stages poses a challenge due to its limited subjective symptoms. While color fundus images offer a cost-effective and noninvasive means of identifying RP, the scarcity of ophthalmologists makes widespread screening impractical in clinical settings. It is generally considered in clinical practice that cases where abnormalities are detectable on fundus images represent an early stage of the disease. Consequently, leveraging AI for RP screening would be highly beneficial. Fundus images contain vast amounts of data, making it challenging to identify subtle abnormalities during routine examinations. Therefore, artificial intelligence can assist in detecting such anomalies within fundus images promptly and accurately, aiding in early intervention. Given the above, there would be a tremendous benefit if AI could be utilized to assist in the early diagnosis of RP.

With the rapid progress of deep learning research, artificial intelligence has been increasingly applied to the diagnosis of various diseases, including ophthalmic disorders [4,5]. Particularly, studies in glaucoma have shown high diagnostic performance and increasing emphasis on explainability, as summarized in a recent systematic review [6]. However, compared with glaucoma, deep-learning applications for RP remain limited, with fewer large-scale studies and less focus on model interpretability and alignment with clinical decision-making. To address this gap, we propose an explainable deep-learning framework for RP classification using fundus images, combining performance evaluation with Grad-CAM-based visualization to provide clinically meaningful insights into model behavior.

Previous studies have explored the application of deep learning to inherited retinal diseases (IRDs), including retinitis pigmentosa (RP), primarily with the aim of predicting causative genes from retinal imaging. Fujinami-Yokokawa et al. demonstrated that deep learning models trained on fundus photography and fundus autofluorescence images could predict prevalent causative genes in IRD with high accuracy, highlighting the feasibility of image-based genetic inference within selected gene subsets [7]. More recently, Pontikos et al. developed Eye2gene, a large-scale, multimodal deep learning framework trained on fundus autofluorescence, infrared imaging, and optical coherence tomography, capable of predicting a wide range of IRD-associated genes and externally validated across multiple international centers [8].

While these studies represent significant advances, they predominantly focus on gene-level classification or genetic prioritization using large, genetically confirmed cohorts and multimodal imaging. In contrast, fewer studies have specifically addressed RP-focused classification using fundus photographs alone, particularly in smaller, retrospective datasets that reflect routine clinical screening settings where genetic information is often unavailable. Our study aims to address this gap by focusing on explainable RP classification based solely on fundus images, emphasizing both diagnostic performance and model interpretability to support real-world clinical decision-making.

Several studies have investigated deep learning-based classification of RP using fundus images. Guo et al. investigated multiple ocular diseases, including RP, using deep transfer learning methods; however, their study did not incorporate cross-validation techniques. [9]. Similarly, Chen et al. reported sensitivity and specificity values of 91.2% and 91.71%, respectively, for the early detection of RP using AI [10]. While these findings demonstrate promising diagnostic performance, they also indicate room for improvement in diagnostic performance.

Beyond classification, AI has been utilized to estimate visual function in RP patients. Nagasato et al. developed a deep learning model using ultra-widefield fundus autofluorescence images to predict visual function parameters such as best-corrected visual acuity and mean deviation in visual fields [11]. The model demonstrated significant correlations between predicted and actual values, suggesting its utility in objectively assessing disease progression. Additionally, Liu et al. applied deep learning to confocal scanning laser ophthalmoscopy images to predict visual impairment in RP patients, achieving robust performance with area under the curve values up to 0.87, thereby providing a proof-of-concept for predicting structure-function correlations based solely on imaging [12]. These studies underscore the potential of AI, particularly deep learning, in enhancing early detection and monitoring of RP through the analysis of fundus images. By leveraging AI, it is possible to identify subtle abnormalities that may be overlooked during routine examinations, facilitating prompt and accurate interventions.

We aim to build upon these prior works by finetuning the existing high-performance deep learning models initially designed and pre-trained for general visual recognition tasks and evaluate accuracy through robust cross-validation procedures to ensure the robustness and generalizability of the finetuned model. Through this approach, we aim to achieve enhanced sensitivity and specificity in the early detection of RP.

## Materials and methods

### Dataset

**Fig 1:**
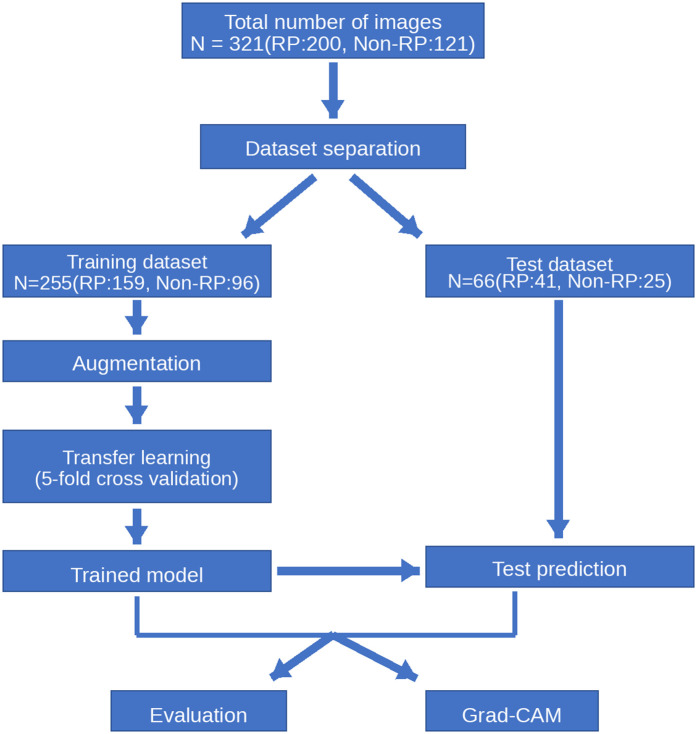
Program flow chart. The dataset containing 321 images was separated into the training and test dataset. We evaluated the models by 5-fold cross-validation on the training dataset and hold-out method using the test dataset.

In this study, an approach for training an AI to predict RP using color fundus images was designed as shown in **Fig 1**. This study was approved by the Ethics Review Committee of Keio University School of Medicine (Approval No. 20210089) and conducted in accordance with the Declaration of Helsinki. Data were accessed for research purposes on September 3, 2022. Written informed consent was waived due to the retrospective nature of the study. The authors did not have access to personally identifiable information during or after data collection. The patient’s personal information was removed from all images prior to the AI analysis.

We collected RP and non-RP images taken at Keio University Hospital during January 2012 through May 2021 using a TRC-50DX retinal camera (Topcon, Japan). The images were generally obtained under pharmacological mydriasis; however, in some cases, insufficient pupil dilation or other clinical factors resulted in reduced image quality. The images were then reviewed by ophthalmologists, and poor-quality images such as significant blur and incorrect viewing angles were removed from the dataset. After that, the images were cropped to the smallest long rectangle that would accommodate the oval shape of the fundus and resized to a (299, 299) size. Since the dataset was relatively small, data augmentation was applied to improve generalization performance. Augmentation was performed online during training rather than by generating a fixed augmented dataset. Specifically, random horizontal flipping and contrast adjustments were applied dynamically to each training batch at runtime. Augmentation was applied only to the training set and not to the validation or test sets to prevent data leakage.

The dataset was randomly divided into training and test sets at a ratio of 4:1 using PyTorch utilities, resulting in 255 images for training and 66 images for testing. This split ratio was chosen to maximize the amount of data available for model training while retaining a sufficient independent test set for unbiased performance evaluation, which is a common practice in medical image classification studies with limited sample sizes. Five-fold cross-validation was further applied within the training set as an internal validation strategy to improve robustness and reduce dependence on a single data split. To prevent data leakage due to bilateral or multiple images from the same patient, all dataset partitions were performed at the patient level. When multiple images, including bilateral images, were available from a single patient, all images from that patient were assigned exclusively to the same subset. Therefore, no patient contributed images to more than one of the training, validation, or test datasets. Cross-validation folds were also generated at the patient level.

Ground truth labels for RP were derived from clinical diagnoses recorded in the electronic medical records and established by board-certified ophthalmologists specializing in retinal diseases at a tertiary referral center. Diagnoses were made based on standard clinical criteria, including patient history, fundus examination findings, visual field testing, and electrophysiological assessments.

Genetic confirmation was not required for inclusion, and therefore genetic diagnoses were not uniformly available. This reflects real-world clinical practice, in which a substantial proportion of patients with clinically diagnosed RP do not undergo genetic testing.

Cases were not stratified by genetic or clinical RP subtypes in the present analysis. The aim of this study was to assess the feasibility of AI-based RP detection using fundus images within a mutation-agnostic, phenotype-driven framework, rather than performing subtype-specific classification. This design is appropriate for screening or diagnostic-assist applications, where detailed subtype information is often unavailable at the time of image acquisition.

Family history information was retrospectively extracted from medical records when available. Among the 107 patients included in this study, family history data were available for 62 patients. A positive family history of retinitis pigmentosa was identified in 22 patients, including autosomal dominant or likely autosomal dominant inheritance (n = 5), autosomal recessive or likely autosomal recessive inheritance (n = 5), X-linked or likely X-linked inheritance (n = 3), and uncertain inheritance patterns (n = 9). The remaining 40 patients with available information were classified as sporadic cases with no reported family history of RP.

Patient demographic data were presented in Table 1.

**Table 1:** Patient demographic characteristics.

		RP	Non-RP
Total		107	94
	Male	56	47
	Female	51	47
Mean age [range]		52.8 [17-87]	54.4 [23-81]

### Model

In this study, deep learning transfer learning techniques with three convolutional neural networks, the VGG16, Inception V3, and Resnet 50 were used. VGG16 is composed of 13 convolutional layers, 5 max-pooling layers, and 3 fully connected layers [13]. InceptionV3 is 42 layers deep and it is using several approaches to reduce the total number of parameters [14]. Resnet 50 has 48 convolutional layers along with 1 maxpool layer and 1 average pool layer [15]. These models were already pretrained on the ImageNet [16], a large-scale public dataset containing general images and their labels, and we subsequently fine-tuned them using our dataset.

Adam optimizer was used for all models, as it is widely adopted in deep learning-based image classification tasks and provides stable and efficient optimization through adaptive learning rates and momentum-based updates, particularly in transfer learning settings. The learning rate was set to 1e-6, which was selected based on preliminary exploratory experiments. Learning rates at different orders of magnitude were tested during initial runs, and 1e-6 showed the most stable convergence behavior, whereas higher values occasionally led to unstable training and lower values resulted in slower convergence. The number of training epochs was set to 200 to ensure sufficient convergence when using this relatively small learning rate. Cross-entropy loss was used as the loss function.

The fundus image dataset comprised of 201 individuals, with 107 individuals diagnosed with RP and 94 individuals classified as non-RP.

### Evaluation

In this study, we adopted a two-stage evaluation process, where we first assessed the performance of the model using cross-validation (CV) and subsequently conducted a final evaluation using a separate test dataset. This approach ensures robustness and reliability in our assessment, as it allows us to validate the generalizability of the finetuned model’s performance beyond the training data. We utilized evaluation metrics, including the receiver operating characteristic (ROC) curve, and its area under the curve (AUC), to assess the performance of the finetuned model.

All models were implemented using PyTorch. Data preprocessing was performed using OpenCV, and model performance was visualized using Matplotlib. Model training and inference were conducted on an Amazon Web Services (AWS) p3 instance equipped with an NVIDIA V100 Tensor Core GPU.

### Visualization

We visualized which parts of the image had the greatest impact on the prediction using Grad-CAM, which is a method for providing an explanation for a given input and its prediction by a CNN-based image recognition model. Ophthalmologists conducted a validity assessment and error analysis of the regions of interest identified based on this map.

### Performance comparison

For further examination of the performance and usefulness of the model, a comparison was made with the performance of ophthalmologists and medical students in classification. The model, four ophthalmologists, and one medical student performed classification using the same 66 images as test data.

## Results

### Image collection

We collected 411 fundus images and excluded 90 images due to poor quality. As a result, our dataset contained 200 RP fundus images from 107 patients and 121 non-RP fundus images from 94 patients. Because both eyes were included when available, some patients contributed more than one image. The collected 321 images consisting of RP and non-RP fundus images were separated into a training set (159 RP and 96 non-RP) and a test set (41 RP and 25 non-RP). The patient demographic characteristics are shown in **Table 1**. Overall, 22 of the 62 patients (35.5%) with available family history information had a positive family history of RP, while 40 patients (64.5%) were sporadic cases.

### 5-fold cross validation

**Fig 2:**
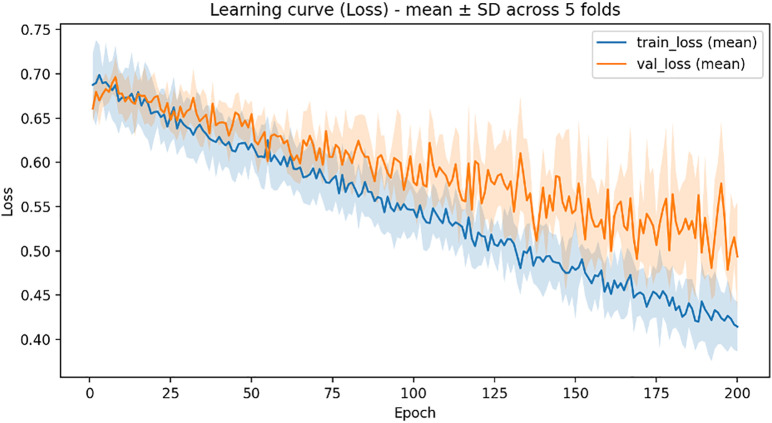
Learning curve of the finetuned InceptionV3 model. Mean training and validation losses across five-fold cross-validation are shown, with shaded areas indicating standard deviation.

We finetuned three deep learning models, and the 5-fold cross validation showed that Inception V3 attained the best performance in accuracy (**Table 2**). For VGG16 the accuracy was 0.749 ± 0.110, for Resnet50 0.883 ± 0.120, and for InceptionV3 0.897 ± 0.078. Fig 2 shows the learning curves of the InceptionV3 model averaged over five cross-validation folds. Training and validation losses decreased consistently throughout training.

**Table 2:** Performance metrics of deep learning models.

Model	Cross Validation Accuracy (± SD)	Test Dataset AUROC, %
VGG16	0.749 ± 0.110	95.21
Resnet50	0.883 ± 0.120	97.85
Inception V3	0.897 ± 0.078	99.32

After training, we conducted testing on the test dataset. Among these, the results of InceptionV3 yielded the best performance, as demonstrated below. As **Tables 2 and 3** shows, the model made wrong predictions only for 2 non-RP images.　For the rest of the images in the dataset (41 RP images and 23 non-RP images), the model made correct prediction (**Table 3**).

**Table 3:** Confusion matrix with Inception V3.

		Predicted Label	
		RP	Non-RP
True Label	RP	41	0
	No RP	2	23

This table shows the performance of the Inception V3 model in classifying data into two categories, RP and Non-RP.

The accuracy and standard deviation (SD) obtained from cross-validation, presented as a percentage. Cross-validation involves dividing the dataset into multiple smaller groups, using each group as a test set to evaluate the model’s generalizability and reliability. The AUROC (Area Under the Receiver Operating Characteristic Curve) value obtained on the test dataset, presented as a percentage. The AUROC value measures how well the model can distinguish between different classes, with higher values indicating better performance.

**Fig 3:**
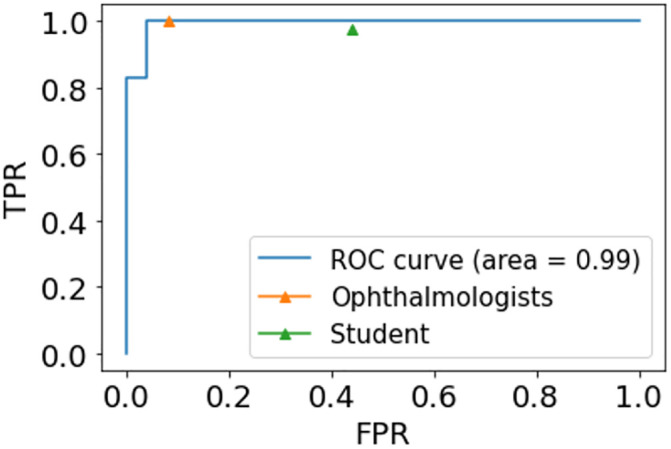
Receiver operating characteristic (ROC) curve of Inception V3 model with plots of the average of ophthalmologists and a student. The ROC curve of Inception V3 model, with performance comparisons to average ophthalmologists and a student, depicted by orange and green triangles respectively. The horizontal axis shows False Positive Rate (FPR), and the vertical axis shows True Positive Rate (TPR).

**Fig 3** shows the performance of the Inception V3 model along with the ophthalmologists and medical student accuracy. Inception V3 scored best in AUROC compared with two other models shown in **Table 2**.

### Visualization

We conducted a Grad-CAM to visualize which pixels the model sees as important in the prediction process for the model based on Inception V3 to confirm the validity of the model to show that it did not rely on features unrelated to RP.

Our analysis revealed that the heatmaps predominantly focus on the peripheral retina, which is consistent with the typical distribution of retinal degeneration in RP. However, in false positive cases, the heatmaps were frequently centered on the macular region, suggesting that the model may misinterpret certain macular patterns as pathological features, leading to misclassification. Notably, the model correctly identified all RP images in the test set. **Fig 5** shows two RP cases with relatively subtle fundus findings that were correctly predicted as RP by the model. Retrospective chart review confirmed that both cases had subtle peripheral degenerative changes and mild attenuation of retinal vessels. Although the peripheral findings were not readily apparent on initial inspection of the color fundus photographs, vascular attenuation was observed around the posterior pole. The Grad-CAM heatmaps highlighted regions around the optic disc and posterior pole, corresponding to the distribution of major retinal vascular structures. These findings raise the possibility that the model relied, at least in part, on secondary features such as vascular attenuation rather than overt peripheral pigmentation. Such indirect cues may be particularly relevant in early or subtle cases where typical fundus findings are not prominent. However, Grad-CAM should be interpreted as a qualitative visualization of model attention and does not by itself establish that the model identified pathognomonic RP features.

**Fig 4:**
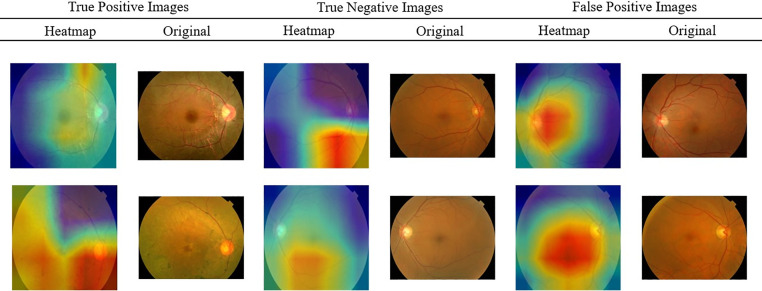
Grad-CAM analysis of misdiagnosed samples. Representative original fundus color images and corresponding Grad-CAM heatmaps are shown for each diagnostic category. The Grad-CAM heatmaps show the regions that contributed significantly to the model’s decision: red and yellow indicate high contribution, blue indicates low contribution.

**Fig 5:**
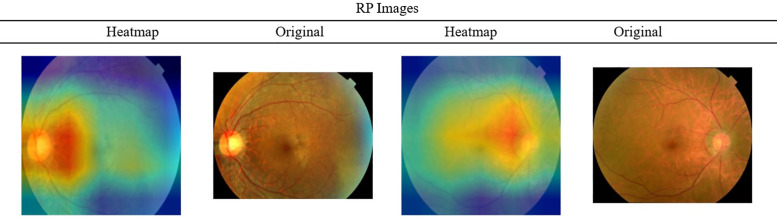
RP cases with subtle fundus findings correctly predicted by the model. Representative original fundus color images and corresponding Grad-CAM heat maps are shown for each diagnostic category.

### Performance comparison with Ophthalmologists and a Medical student

As shown in Table 4 and Fig 3, the classification performance of the finetuned InceptionV3 model was compared with that of ophthalmologists and a medical student. The model achieved performance levels comparable to those of the ophthalmologists and superior to those of the medical student, with an accuracy of 96.97% on the test set. While the confidence intervals were relatively wide due to the limited test sample size, the point estimates consistently supported similar classification performance between the model and the ophthalmologists. Furthermore, our results are at least comparable to and slightly better than those reported in previous studies [9,10], strengthening the validity of our finetuning approach.

**Table 4:** Performance comparison of the finetuned InceptionV3 model, ophthalmologist, and medical student.

	Accuracy, %	Recall, %	Specificity, %	Precision, %
Ophthalmologist 1	95.45	92.68	100	100
Ophthalmologist 2	98.48	97.56	100	100
Ophthalmologist 3	96.97	95.12	100	100
Ophthalmologist 4	96.97	95.12	100	100
Ophthalmologists Mean	96.97	95.12	100	100
Medical student	81.82	73.17	96.00	96.77
Finetuned InceptionV3 (95% CI)	96.97(89.48-99.63)	100 (91.40-100.00)	92 (73.9- 99.02)	95.35 (84.19-99.43)

The table displays accuracy, recall, specificity, and precision for four ophthalmologists (labeled 1–4), their mean values, a medical student, and a machine learning model. Confidence intervals were estimated by the Clopper-Pearson exact method.

## Discussion

### Performance

RP is a retinal degenerative disease that can lead to loss of sight. Early detection is important in terms of reducing the psychological burden of possible future disabilities even if there is no effective treatment for now. This study addressed the primary research question of whether deep learning-based classification models could improve the diagnostic performance for retinitis pigmentosa (RP) using fundus images. We experimented with VGG16, Resnet50, and Inception V3 to construct the model. As a result, Inception V3 reached the highest accuracy. Because the dataset was relatively small, we used 5-fold cross-validation as an internal validation step to reduce dependence on a single train-test split, enabling a more robust comparison than prior studies.

Since Resnet50 and Inception V3 are improved networks of VGG16, they performed better than VGG16. For an image classification task like this, features can exist anywhere in the image at various sizes. That is why choosing a kernel size is difficult. Inception V3 is designed to capture image features at multiple spatial scales by applying filters of varying sizes in parallel, which is advantageous for fundus image classification where pathological features may appear at different locations and sizes. This architectural characteristic likely contributed to the superior performance observed in our study. In comparison to the prior study conducted by Chen et al., which reported sensitivity and specificity values of 91.2% and 91.71%, respectively, we achieved higher sensitivity and specificity rates in our classification model. This improvement underscores the effectiveness of our approach in accurately identifying individuals with RP, thereby highlighting the potential clinical utility. To more clearly position our contribution relative to prior work, Table 5 summarizes a comparative overview of recent deep learning-based studies for RP classification [9,10,17–23]. In contrast to previous studies, which primarily relied on a single train–test split and reported limited model interpretability, our approach combines five-fold cross-validation with Grad-CAM–based visualization. This design enables both more robust performance estimation and clinically interpretable model behavior. Notably, our model achieved higher sensitivity and comparable or improved specificity despite a relatively small dataset, supporting the novelty of integrating rigorous internal validation with explainable decision-making in RP detection.

**Table 5:** Comparison of representative deep learning–based studies for retinitis pigmentosa classification.

Study (Year)	Task	Dataset	Model & Method	Validation	Performance	Explainability
Masumoto et al. (2019)	Binary classification (RP vs normal) using UWF fundus images	373 UWF images (150 RP, 223 normal), single-center	VGG-16 CNN (ImageNet transfer learning)	5-fold cross-validation	AUC 0.998–1.00; Sens 99–100%; Spec 99–99.5%	Grad-CAM
Chen et al. (2021)	Binary classification (RP vs normal) using color fundus photos	1,670 fundus images (935 RP, 735 normal), Taiwan IRD cohort	Xception CNN (transfer learning)	5-fold cross-validation + independent test set	AUROC 96.7%; Acc 96.0%; Sens 95.7%	Grad-CAM
Guo et al. (2021)	Multi-class classification (5 eye diseases incl. RP + normal)	250 fundus images (5 classes), public dataset	MobileNetV2 CNN (transfer learning)	Five independent runs	Acc 96.2%; Sens 90.4%	Grad-CAM
Rieck et al. (2025)	Multi-class classification (9 retinal diseases + healthy)	16,242 fundus images, public multi-disease dataset	ResNet-50-based CNN with tailored preprocessing	5-fold cross-validation	Balanced accuracy ~82.5%	Saliency maps
Duan et al. (2025)	Multi-class classification (16 fundus diseases incl. RP)	10,612 UWF images from two hospitals (multi-center)	DenseNet-121 + XGBoost hybrid model	Internal validation + external test set	AUC > 0.89 (rare diseases); Acc > 99% for RP	Grad-CAM
Jafarbeglou et al. (2025)	Multi-class classification (RP vs STGD vs healthy)	391 cases with CFP + IR images	MobileNetV2 (dual-input multimodal CNN)	Train–test split	Accuracy 96.3% (CFP + IR)	Grad-CAM
Karimi et al. (2025)	Multi-class classification (RP, STGD, healthy)	5,844 unlabeled + 782 labeled fundus images	EfficientNet-B1 with self-supervised pretraining	Train–test split	Accuracy 98.15%; AUC 99.68%	Grad-CAM
Nagai et al. (2026)	RP detection and visual prognosis prediction	Large multi-center RP fundus dataset + clinical data	EfficientNet-B4 + survival analysis model	Patient-level train–test split	Diagnosis AUC 0.94; Prognosis AUC ~ 0.8–0.9	SHAP
Hwang et al. (2025)	Inheritance pattern classification (autosomal vs X-linked RP)	Small RP cohort with synthetic data augmentation	Vision Transformer + VAE augmentation	Cross-validation	AUC improved from 0.67 to 0.79	Not reported
Our study	Binary classification (RP vs normal) using color fundus photos	321 fundus images (200 RP, 121 normal), single-center	Inception V3 CNN (transfer learning)	5-fold cross-validation + independent test set	AUROC 99.32%; Acc 96.97%; Sens 100%	Grad-CAM

More broadly, recent AI research in inherited and rare retinal diseases has highlighted several key challenges that are directly relevant to the present study, including limited sample size, external validation, multimodal integration, and clinical interpretability [24,25]. Data scarcity is a fundamental obstacle in rare retinal diseases, and conventional transfer learning alone may not be sufficient to ensure robust generalization. Recent work on macular hole detection and segmentation using large multimodal generative models for synthetic augmentation demonstrated a potential strategy for addressing severe data scarcity in fundus photography, although the authors emphasized that the findings should be interpreted as exploratory and that larger validation studies are needed before clinical deployment [26]. Public fundus datasets, such as JSIEC, may also provide useful preliminary resources for external testing, but differences in image acquisition, diagnostic labeling, and disease-specific sample size can limit direct generalizability [27]. In parallel, multimodal approaches such as Eye2Gene have illustrated the potential value of integrating fundus autofluorescence, infrared reflectance, and OCT images for inherited retinal disease phenotyping and genetic diagnosis [8]. Recent reviews of AI applications in inherited retinal diseases have similarly emphasized that robust external validation, multimodal integration, and explainable or clinically interpretable frameworks remain essential for clinical translation [24,25]. In this context, our study contributes to RP detection using color fundus photographs with internal cross-validation and Grad-CAM–based visualization, while also highlighting the need for larger multicenter datasets, multimodal approaches, and prospective external validation in future work.

Our Grad-CAM analysis, as shown in Fig 4, provides insight into the image regions that most strongly influenced the model’s classification decisions. In both true positive and true negative cases, the model predominantly attends the peripheral retina. In true positive images, this focus corresponds to peripheral pigmentation and degenerative changes that are characteristic of RP, reflecting the typical centrifugal progression of the disease. In true negative cases, the heatmaps similarly highlight peripheral regions; however, these regions correspond to normal fundus features rather than pathological changes. This suggests that the model actively evaluates the absence of peripheral degenerative findings when deciding not to predict RP, consistent with the diagnostic strategy used by ophthalmologists.

In contrast, in the two false-positive cases, the program primarily concentrated on the central macular area of the image. The probable cause for this misfocuses might arise from the misinterpretation of the macular tigroid pattern as retinal degenerative changes. Additionally, these errors could stem from insufficient data. These findings underscore the necessity for further exploration into the factors contributing to misfocusing, particularly in distinguishing macular patterns from degenerative changes, to refine the accuracy of the classification model. In summary of the visualization, it can be inferred that the model predominantly directs attention to pigmentation in the periphery and the macula for prediction, mirroring the approach of ophthalmologists. These findings indicate that the model’s decision-making process is largely clinically interpretable, while also highlighting the need for further refinement to reduce misclassification caused by normal macular patterns. In the current study, part of the model evaluation already involved review by an ophthalmologist, particularly for misclassified cases, to assess whether the Grad-CAM attention regions were clinically plausible. This preliminary expert-in-the-loop analysis supported that most true positive heatmaps corresponded to peripheral degenerative changes consistent with RP pathology. However, more systematic validation with medical experts is required. Future studies should include prospective human-AI comparison experiments, inter-rater agreement analysis, and structured review of false-positive and false-negative cases to quantitatively assess how AI assistance impacts diagnostic accuracy, confidence, and decision-making time in clinical practice. Going back to the performance of the model was as high as that of the ophthalmologists’ diagnoses. The reason for the high performance despite the relatively small amount of data is thought to be that the abnormal findings were relatively easy to detect.

Taken together, these findings demonstrate that our deep learning model not only achieved high diagnostic performance through rigorous cross-validation but also exhibited clinically interpretable attention patterns, fulfilling the primary objectives of this study.

From a clinical translation perspective, the Grad-CAM-based visualization is particularly important for building trust in real-world deployment. Similar to recent clinically oriented deep learning systems for fundus image analysis our approach provides visual evidence that the model focuses on pathophysiologically relevant regions, namely the peripheral retina [10].This interpretability supports the potential use of the proposed model as a decision-support or triage tool in non-specialist or screening settings, rather than as a standalone diagnostic system. In future implementations, such a model could assist ophthalmologists by flagging suspected RP cases for further expert review, thereby improving workflow efficiency and early case detection.

Beyond interpretability, practical deployment in real-world clinical settings remains a critical challenge for AI-based fundus image analysis. Recent clinically oriented studies have demonstrated the feasibility of deploying AI-based fundus image analysis systems in routine practice, including workflow integration, prospective evaluation, and interaction with clinicians (e.g., IEEE Access, 2024). In line with these approaches, the proposed model is intended to support RP screening by flagging suspected cases during routine fundus examinations, particularly in non-specialist or high-volume settings. Before clinical deployment, several steps are required, including multicenter external validation, evaluation across different fundus cameras and imaging protocols, and prospective assessment of clinical impact on diagnostic accuracy and workflow efficiency. Integration with existing picture archiving and communication systems and presentation of Grad-CAM-based visual explanations will also be critical to ensure usability and clinician trust. By positioning the model within a decision-support framework rather than an autonomous diagnostic system, our approach aligns with recent successful examples of real-world AI deployment in ophthalmology and addresses key barriers that have limited clinical adoption of AI-based models.

Although the learning curves showed stable convergence without divergence of validation loss, a modest gap between training and validation loss remained, indicating a mild generalization gap rather than severe overfitting. Underfitting was unlikely, as both losses decreased steadily throughout training. This limitation is likely attributable to the relatively small, single-center dataset. Future work using larger, multi-institutional datasets, as well as additional regularization strategies and external validation, will be essential to further improve generalizability.

The table summarizes differences in task definition, dataset composition, model architecture, validation strategy, reported performance metrics, and model interpretability. Direct comparison of performance across studies should be interpreted with caution due to differences in problem settings and evaluation protocols.

### Limitations and future research

Our work has several limitations. First, the number of images used in the study was limited, reflecting the difficulty of constructing large datasets for rare retinal diseases such as RP. Although the model showed high performance in the present dataset, further accumulation of training and validation data is necessary to improve robustness and clinical applicability. Second, this model was designed to detect the presence or absence of RP and cannot determine the causative gene, disease stage, severity, or prognosis. Recent studies have proposed grading systems for RP severity using clinical findings, visual acuity, perimetry, and OCT measurements. For instance, Smith et al. developed a grading system that correlated clinical appearance with quantitative measures such as best-corrected visual acuity and mean deviation from perimetry, demonstrating its potential to predict disease progression [28]. Incorporating such grading systems into AI models may enable future systems not only to detect RP but also to assess disease severity and support clinical decision-making. Third, all images used in this study were obtained from a single institution, Keio University Hospital, and no independent external validation cohort was included. Therefore, the generalizability of the model to other institutions, imaging devices, ethnic populations, and clinical settings remains uncertain. This limitation is particularly important for RP because the disease shows substantial heterogeneity in age at onset, severity, fundus appearance, disease stage, and genetic background. Real-world deployment would require prospective validation using multicenter datasets, including images acquired from different fundus cameras and imaging protocols. Robustness against variations in image quality, illumination, and device-specific artifacts must also be systematically evaluated before clinical integration. In addition, workflow-level considerations, such as automatic preprocessing, compatibility with picture archiving and communication systems, and real-time inference performance, will be critical for practical implementation in outpatient or screening environments. Future studies should therefore focus on multicenter external validation, evaluation across disease stages and genetic subtypes, integration of multimodal imaging data, and prospective assessment of clinical utility. Addressing these issues may lead to more robust and clinically useful AI tools for the early detection and management of RP.

## Conclusion

This study reports the development of an AI to diagnose RP from fundus images of Japanese patients. It achieved results comparable to ophthalmologists’ diagnoses. Additionally, Grad-CAM suggested that the model attended to clinically relevant fundus regions, including the peripheral retina and posterior pole, supporting the potential interpretability of the model. We also discovered that the model may mistakenly identify macular patterns as indicative of RP. The results of this study suggest the usefulness of using deep learning for the automatic determination of RP or tools to reduce the burden on ophthalmologists.

## Abbreviation:

AI: Artificial Intelligence
AUC: Area Under the Curve
FPR: False Positive Rate
Grad CAM: gradient-weighted class activation mapping
ROC: Receiver Operating Characteristic
RP: retinitis pigmentosa
TPR: True Positive Rate
