# Supplementary material for: Fundus image analysis of retinitis pigmentosa using artificial intelligence
Source: PLoS One. 2026 Jul 24;21(7):e0354452. doi: 10.1371/journal.pone.0354452 (PMC13399350; doi:10.1371/journal.pone.0354452)
Supplement: Attachment — Submitted filename: response_to_reviewers unmarked.docx [file pone.0354452.s002.docx]

**Response to reviewer comments**

Dear Dr. Vahid Mansouri

We would like to thank you for handling our manuscript and for providing us with the opportunity to revise it. We are also grateful to all reviewers for their careful reading of our work and for their constructive and insightful comments, which have helped us to substantially improve the quality, clarity, and rigor of the manuscript.

We have carefully considered each comment and have revised the manuscript accordingly. All changes have been made with the aim of addressing the reviewers’ concerns in a transparent manner.

Below, we provide a point-by-point response to each comment raised by the reviewers. Reviewer comments are reproduced with a colored background for clarity, followed by our responses. Where relevant, revised or newly added text from the main manuscript is shown in italics.

# Journal Requirements

1. Please ensure that your manuscript meets PLOS ONE's style requirements, including those for file naming. The PLOS ONE style templates can be found at
<https://journals.plos.org/plosone/s/file?id=wjVg/PLOSOne_formatting_sample_main_body.pdf>
and
<https://journals.plos.org/plosone/s/file?id=ba62/PLOSOne_formatting_sample_title_authors_affiliations.pdf>

**2.** In the online submission form, you indicated that “The dataset is not publicly available due to its containing information that could compromise the privacy of research participants but are available from the corresponding author upon reasonable request.”

All PLOS journals now require all data underlying the findings described in their manuscript to be freely available to other researchers, either
1. In a public repository,
2. Within the manuscript itself, or
3. Uploaded as supplementary information.

This policy applies to all data except where public deposition would breach compliance with the protocol approved by your research ethics board.

If your data cannot be made publicly available for ethical or legal reasons (e.g., public availability would compromise patient privacy), please explain your reasons on resubmission and your exemption request will be escalated for approval.

**Response:**
 Thank you for this important clarification regarding the PLOS data availability policy. The primary data underlying this study consist of retinal fundus images obtained from human participants. Due to the unique and potentially identifiable nature of retinal vascular patterns, public deposition of these images could compromise patient privacy, even after de-identification. Therefore, public sharing of the image dataset is not permitted under the protocol approved by the Ethics Committee of Keio University School of Medicine and is restricted for ethical reasons. We respectfully request an exemption from public data deposition for the image dataset based on these ethical restrictions. To ensure reproducibility of our findings, we confirm that all information necessary to replicate the analyses, including the software libraries, model architectures, training procedures, cross-validation strategy, and hyperparameters, has been fully described in the Methods section of the manuscript. In addition, the summary results and evaluation metrics are provided within the manuscript. The Data Availability Statement has been revised accordingly to reflect these points.

3. We note that you have indicated that there are restrictions to data sharing for this study. PLOS only allows data to be available upon request if there are legal or ethical restrictions on sharing data publicly.

For more information on unacceptable data access restrictions, please see
<http://journals.plos.org/plosone/s/data-availability#loc-unacceptable-data-access-restrictions>

Before we proceed with your manuscript, please address the following prompts:

a) If there are ethical or legal restrictions on sharing a de-identified data set, please explain them in detail (e.g., data contain potentially identifying or sensitive patient information, data are owned by a third-party organization, etc.) and who has imposed them (e.g., a Research Ethics Committee or Institutional Review Board, etc.). Please also provide contact information for a data access committee, ethics committee, or other institutional body to which data requests may be sent.

b) If there are no restrictions, please upload the minimal anonymized data set necessary to replicate your study findings to a stable, public repository and provide us with the relevant URLs, DOIs, or accession numbers.

For a list of recommended repositories, please see
<https://journals.plos.org/plosone/s/recommended-repositories>

You also have the option of uploading the data as Supporting Information files, but we would recommend depositing data directly to a data repository if possible.

We will update your Data Availability statement on your behalf to reflect the information you provide.

**Response:**
Thank you for this comment. The dataset consists of retinal fundus images from human subjects, which may retain potentially identifying features even after de-identification. Therefore, public data sharing is ethically restricted to protect patient privacy. In addition, explicit informed consent for public data sharing was not obtained from the participants at the time of data collection. Therefore, unrestricted public release of the raw image data is not permitted. The Data Availability Statement has been revised accordingly. Requests for data access may be directed to the Ethics Committee of Keio University School of Medicine or the corresponding author.

4. Please amend your Ethics Approval number to ensure that it matches in both the Manuscript and the Details page.

**Response:**
 Thank you for pointing this out. We carefully re-checked the Ethics Approval number in both the manuscript and the online submission details page and confirm that the approval number is now consistent in both locations.

5. If the reviewer comments include a recommendation to cite specific previously published works, please review and evaluate these publications to determine whether they are relevant and should be cited. There is no requirement to cite these works unless the editor has indicated otherwise.

**Response:**
 We carefully reviewed the previously published works recommended by the reviewer and evaluated their relevance to the present study. Based on this assessment, we have cited the publications that were directly relevant and appropriate to the scope of our research. The manuscript has been revised accordingly.

# Reviewer #1

1. It would be better to highlight the main research questions in the introduction section, and describe in the Discussion section, how you fulfilled those in the study, by referring to the applied methods and the obtained results.

**Response:**
 Thank you for this helpful suggestion. We have revised the Introduction section to more clearly highlight the main research questions of the study on page 7, lines 114-121. In addition, we have expanded the Discussion section to explicitly address how these research questions were fulfilled, with reference to the applied methods and the obtained results on page 28, lines 411-414. These revisions were made to improve the clarity and coherence of the manuscript.

2. Introduction - state the gaps in the latest literature including <https://doi.org/10.1016/j.imu.2025.101644> and how the proposed research aims to address them.

**Response**:

We thank the reviewer for this helpful suggestion. We have revised the Introduction to reference a recent systematic review on deep-learning–based glaucoma identification and to highlight the gap between the well-developed glaucoma literature and the relatively limited, less explainable deep-learning research in retinitis pigmentosa on pages 4-5, lines 64-73. We also clarified how our proposed framework addresses this gap by emphasizing both diagnostic performance and Grad-CAM–based interpretability.

3. Provide a rationale for the chosen hyperparameters: learning rate (0.000001), number of epochs (200), and the use of the Adam optimizer. Were other values tested, and what was the impact on performance?

**Response**:

We thank the reviewer for this helpful comment. We have clarified the rationale for the selected hyperparameters in the Methods section on pages 12-13, lines 193-202, including the learning rate, number of epochs, and the use of the Adam optimizer. Other learning rates were explored in preliminary experiments, and the selection was based on observed training stability and convergence behavior.

The following text has been added to the manuscript:

*Adam optimizer was used for all models, as it is widely adopted in deep learning–based image classification tasks and provides stable and efficient optimization through adaptive learning rates and momentum-based updates, particularly in transfer learning settings. The learning rate was set to 1e-6, which was selected based on preliminary exploratory experiments. Learning rates at different orders of magnitude were tested during initial runs, and 1e-6 showed the most stable convergence behavior, whereas higher values occasionally led to unstable training and lower values resulted in slower convergence. The number of training epochs was set to 200 to ensure sufficient convergence when using this relatively small learning rate. Cross-entropy loss was used as the loss function.*

4. Proofread for typographic errors. Eg. Line 152 mentions that Inception V3 attained the best performance in accuracy in Table 1, but Table 1 is the Patient demographic characteristics. The performance metrics are in Table 2. Please correct the in-text reference from Table 1 to Table 2 for the cross-validation results.

**Response:**
 Thank you for pointing out this typographical error. We have carefully proofread the manuscript and corrected the in-text reference in Line 258. The performance metrics of the models, including the accuracy achieved by Inception V3, are now correctly referenced to Table 2, which presents the cross-validation results, instead of Table 1 (patient demographic characteristics). The manuscript has been revised accordingly.

5. The Abstract states the dataset contained 200 RP images and 121 non-RP images. The Results section mentions 200 RP images from 107 patients and 121 non-RP images from 94 patients. It should be confirmed that the study uses 200 RP images and 121 Non-RP images (Total 321 images) for the classification task, and that some patients contributed multiple images.

**Response:**
 Thank you for this important comment. We confirm that the classification task consistently used 200 RP images and 121 non-RP images (total 321 images) throughout the study. As noted in the Results section, these images were obtained from 107 RP patients and 94 non-RP patients, and some patients contributed images from both eyes, resulting in multiple images per patient.

To avoid confusion, we have revised the Results section (page 15, lines 236-239) to explicitly state that the dataset includes bilateral eye images from certain patients, while the total number of images used for classification remains unchanged.

6. Describe the implementation details such as used libraries and the hardware specification (CPU/ GPU)

**Response**:

We thank the reviewer for this helpful suggestion. In response, we have updated the Materials and Methods section (page 13, lines 213-216) to explicitly describe the implementation environment. The revised text now specifies the software libraries used for preprocessing, model implementation, and visualization, as well as the hardware configuration used for training and inference as follows:

*All models were implemented using PyTorch. Data preprocessing was performed using OpenCV, and model performance was visualized using Matplotlib. Model training and inference were conducted on an Amazon Web Services (AWS) p3 instance equipped with an NVIDIA V100 Tensor Core GPU.*

7. Include more evaluation metrics such as confusion matrix and the learning curves (training and validation/ accuracy and loss)
8. Discuss the issues and limitations (such as overfitting/ underfitting) in the training model and how you can address those.

Response: We thank the reviewer for these constructive comments. In response, we have added the learning curves of the fine-tuned InceptionV3 model to the main text as Figure 2, showing the mean training and validation losses across five-fold cross-validation. Confusion matrix is shown as Table 3.

We have also expanded the Discussion section (page 30, lines 440-446) to explicitly address potential overfitting, its likely cause, and potential solution, as follows:

*Although the learning curves showed stable convergence without divergence of validation loss, a modest gap between training and validation loss remained, indicating a mild generalization gap rather than severe overfitting. Underfitting was unlikely, as both losses decreased steadily throughout training. This limitation is likely attributable to the relatively small, single-center dataset. Future work using larger, multi-institutional datasets, as well as additional regularization strategies and external validation, will be essential to further improve generalizability.*

9. For all the results figures and tables, clearly explain the observations and the decisions that can be taken by those observations. Eg. Figure 3 -, clarify what the Grad-CAM heatmap is highlighting. Since the image is correctly classified as Non-RP, the heatmap indicates the regions that most contributed to the model's decision not to predict RP. Discuss whether these "important pixels" align with expected normal fundus features, or if they point to regions the model actively avoids focusing on.

**Response**:

We thank the reviewer for this valuable comment. We have revised the Discussion section to explicitly describe what the Grad-CAM heatmaps highlight and how these regions contribute to the model’s decisions on pages 26-27, lines 377-402. We now clarify that in both true positive and true negative cases the model focuses on peripheral regions, reflecting evaluation of the presence or absence of typical peripheral degenerative changes. We also explain that false positive cases arise from incorrect attention to central macular patterns. These revisions clarify the interpretability of Figure 4 and the clinical relevance of the highlighted regions.

10. Include a comparison table in the discussion, stating the new features and results of the proposed model, in comparison to the latest such studies. By that, justify your novel contribution.

**Response:**
 We thank the reviewer for this valuable suggestion. To clearly highlight the novelty of our work, we have added a comparison table (Table 5) in the Discussion section, summarizing recent RP classification studies in terms of model architecture, validation strategy, performance, and interpretability. This table demonstrates that our proposed model uniquely combines rigorous cross-validation with Grad-CAM–based explainability while achieving competitive or superior diagnostic performance, thereby clarifying our novel contribution.

11. Discuss the future work and possibility of deploying the proposed model as a real world application in clinical settings such as in <https://doi.org/10.1109/ACCESS.2024.3359698>. Because most of the AI based models are not implemented for actual practice.

**Response:**
 Thank you for this important suggestion. We have added a new paragraph in the Discussion section addressing the future work and real-world deployment potential of our model, in reference to recent clinically oriented studies. We clarify that our model is intended as a decision-support tool for RP screening and triage rather than a standalone diagnostic system. We also discuss key requirements for clinical translation, including multicenter external validation, robustness across imaging devices, workflow integration, and prospective evaluation. In addition, we highlight the role of Grad-CAM–based explainability in improving clinical trust and adoption.

Added text in the Discussion

Lines:100-113

Beyond interpretability, practical deployment in real-world clinical settings remains a critical challenge for AI-based fundus image analysis. Recent clinically oriented studies have demonstrated the feasibility of deploying AI-based fundus image analysis systems in routine practice, including workflow integration, prospective evaluation, and interaction with clinicians (e.g., IEEE Access, 2024). In line with these approaches, the proposed model is intended to support RP screening by flagging suspected cases during routine fundus examinations, particularly in non-specialist or high-volume settings. Before clinical deployment, several steps are required, including multicenter external validation, evaluation across different fundus cameras and imaging protocols, and prospective assessment of clinical impact on diagnostic accuracy and workflow efficiency. Integration with existing picture archiving and communication systems and presentation of Grad-CAM-based visual explanations will also be critical to ensure usability and clinician trust. By positioning the model within a decision-support framework rather than an autonomous diagnostic system, our approach aligns with recent successful examples of real-world AI deployment in ophthalmology and addresses key barriers that have limited clinical adoption of AI-based models.

12. What is the possibility of validating this approach with medical experts.

**Response:**
 Thank you for this comment. We have clarified in the manuscript that part of the model evaluation already involved review by an ophthalmologist, particularly for misclassified cases, to assess whether the Grad-CAM attention regions were clinically plausible on page 27, lines 398-402. We have also added a discussion of future expert-in-the-loop validation (page 35, lines 482-489), including prospective human–AI comparison studies, inter-rater agreement analysis, and systematic review of false-negative and false-positive cases. These steps are planned as the next phase to strengthen the clinical reliability and applicability of the proposed approach.

# Reviewer #2

1.The manuscript stated the dataset was separated into training and test sets but failed to specify the ratio, rationale, and method for this split. Furthermore, the exact size of the training set after augmentation was not provided.
Authors must explicitly detail the exact proportion of training and test sets, including the augmentation samples.

**Response**：

Thank you for this comment. We have revised the Methods section to explicitly describe the dataset split strategy and data augmentation procedure on page 9, lines 139-153. The dataset was randomly divided into training and test sets at a ratio of 4:1 using PyTorch, and the rationale for this split is now stated. Data augmentation was performed online during training, and therefore no fixed augmented dataset size exists; this has been clarified in the manuscript. We have also explained that five-fold cross-validation was applied within the training set as an internal validation strategy.

2.The manuscript only displayed Grad-CAM heatmaps for misdiagnosed samples, failing to contrast the regions of focus between correctly and incorrectly diagnosed cases.
Authors should provide visual evidence demonstrating that heatmaps of true positive samples indeed concentrate on the peripheral retina.

**Response:**
 Thank you for this comment. We have added Grad-CAM heatmaps for correctly classified RP samples and included them in Figure 4. These examples show that the model predominantly focuses on the peripheral retina in true positive cases, in contrast to misdiagnosed samples. A corresponding explanation has been added to the Results/Discussion section.

3.There was no statistical comparison, like confidence intervals and p-values, to quantify the differences between models and support the claim that the model's performance was comparable to ophthalmologists.

**Response**: We thank the reviewer for this important and constructive comment regarding the statistical comparison. In response, we have revised the Results section to include 95% confidence intervals for the model’s performance metrics (accuracy, sensitivity, specificity, and precision), thereby explicitly quantifying the uncertainty associated with the estimated performance. The corresponding values and their interpretation are now shown in Table 4 and Results:

Lines: 327-335

*As shown in Table 4 and Figure 3, the classification performance of the finetuned InceptionV3 model was compared with that of ophthalmologists and a medical student. The model achieved performance levels comparable to those of the ophthalmologists and superior to those of the medical student, with an accuracy of 96.97% on the test set. While the confidence intervals were relatively wide due to the limited test sample size, the point estimates consistently supported similar classification performance between the model and the ophthalmologists.*

We did not perform formal hypothesis testing (e.g., equivalence or non-inferiority tests) for the comparison between the model and ophthalmologists, as the evaluation was based on a relatively small test set and such tests were not pre-specified in the original study design. Conducting post hoc hypothesis testing under these conditions could risk overinterpretation. Instead, we focused on confidence interval-based estimation, which we believe provides a more appropriate and transparent characterization of model performance in this setting. This allows readers to assess both the point estimates and their associated uncertainty without relying on dichotomous conclusions based on p-values. Accordingly, the revised text emphasizes that while the point estimates indicate similar performance between the model and ophthalmologists, definitive conclusions regarding statistical equivalence cannot be drawn from the present dataset, and larger-scale validation studies will be required.

We appreciate the reviewer’s suggestion, as it has helped us to present the comparison between the model and ophthalmologists in a more statistically rigorous and transparent manner, and to more clearly communicate the uncertainty and limitations of the current evaluation.

4.The patient demographic description was overly simplistic. For an inherited disease like RP, the family history is critical. Its absence from the dataset description raised questions about the clinical representativeness of the RP patients.
Authors should supplement Table 1 to include the proportion of patients with family history of RP. If this information is unavailable from the retrospective records, it should be explicitly stated and discussed in the manuscript.

**Response**：

We thank the reviewer for this important comment. We agree that family history is a critical clinical factor in inherited diseases such as retinitis pigmentosa. While family history information was not consistently available for all patients due to the retrospective nature of the study, we have revised the manuscript to explicitly report the proportion of patients with available family history data and the distribution of familial and sporadic cases in the Methods and Results sections on pages 10-11, lines 169-176 and on page 16, lines 242-244.

5.The manuscript noted the use of cross-validation as a distinction from some prior work but failed to explain why this was methodologically critical. The purpose and advantages of CV was not fully articulated.
In the Discussion, authors must explicitly state the role of k-fold cross-validation.

**Response**: We appreciate the reviewer’s insightful comment. We have revised the Discussion to explicitly state the methodological role of 5-fold cross-validation. We now clarify that cross-validation was used as an internal validation strategy to reduce dependence on a single data split, which is particularly important for robust evaluation in small datasets.

Lines: 352-255

*Because the dataset was relatively small, we used 5-fold cross-validation as an internal validation step to reduce dependence on a single train-test split, enabling a more robust comparison than prior studies.*

6.Description of the data in Table 3 in line 158 was inconsistent with the 1 content.

Furthermore, several inconsistencies and errors reduce the manuscript's polish.

1.There are textural errors in line 24, line 25, line 229, line 249.

2.The legend for Table 2 was incorrectly positioned in line 161.

**Response**:

Thank you for pointing out these issues. We have carefully revised the manuscript to correct all identified inconsistencies and typographical errors. The description of the data in Table 3 has been revised to ensure consistency with the table content. Textual errors throughout the manuscript have been corrected, and the legend for Table 2 has been repositioned to the appropriate location. In addition, the entire manuscript was rechecked to improve clarity, consistency, and overall polish.

# Reviewer #3

1. The sample size and diversity of the training dataset need clarification. The author should specify many patients and images were included. Whether images from multiple centers included to ensure generalizability? How was the dataset split for training, validation, and testing? Were external validation cohorts used?

**Response:**
 Thank you for this comment. We have clarified the sample size and dataset composition in the Methods section (page 9, line 147-148) by specifying the number of patients and images included. All images were obtained from a single center. The dataset was randomly split into training and test sets with the pre-specified ratio. Validation set was derived from training dataset during cross-validation. No external validation cohort was used in this study, which we have acknowledged as a limitation in the Discussion on page 29, lines 431-434.

2. The establishment of ground truth labels is critical but insufficiently described. Who determined the ground truth labels for RP (single expert, consensus panel)? Was there a genetic diagnosis to confirm RP? Did you consider different subtypes of RP?

**Response**:

We appreciate the reviewer’s important comment regarding the establishment of ground truth labels.

In this study, the ground truth labels for retinitis pigmentosa were based on the clinical diagnosis documented in the medical records, made by board-certified ophthalmologists specializing in retinal diseases at a tertiary referral center. The diagnosis was determined based on standard clinical criteria, including patient history, fundus examination, visual field testing, and electrophysiological findings.

Genetic confirmation was not required for inclusion, and therefore genetic diagnoses were not uniformly available. This reflects real-world clinical practice, where a substantial proportion of patients with clinically diagnosed RP do not have a confirmed genetic diagnosis.

Regarding RP subtypes, we did not stratify cases by genetic or clinical subtype in the present analysis. The objective of this study was to evaluate the feasibility of AI-based RP detection using fundus images under a mutation-agnostic, phenotype-driven framework, rather than subtype-specific classification. We believe this approach is appropriate for a screening or diagnostic-assist setting, where detailed subtype information is often unavailable at the time of image acquisition.

We have clarified these points in the Methods section to improve transparency on page 10, lines 159-168.

3. More details about the imaging needed. What retina scanner was used to acquire the images, where the pupils dilated on acquisition? Bad quality images are mentioned. What was the criteria to exclude bad quality images? Were any other preprocessing step performs like normalisation or cropping on the images?

**Response**:

We thank the reviewer for the detailed and constructive comment regarding the imaging methodology. We have revised the Methods section to provide additional details on image acquisition and preprocessing.

Specifically, we now state that fundus images were acquired using a TRC-50DX retinal camera (Topcon, Japan) and were generally obtained under pharmacological mydriasis. We also clarify that image quality varied in some cases due to insufficient pupil dilation or other clinical factors.

We have further specified the criteria used to exclude poor-quality images, including significant blur, incorrect angle of view, and severe artifacts, all of which were assessed by ophthalmologists. In addition, we have clarified the preprocessing steps, including cropping to the fundus region, resizing, pixel normalization, and online data augmentation applied only to the training set to avoid data leakage.

Those are on pages 8, lines 133-138.

4. More technical details about the AI model needed. Were pre-trained models like a foundation model used, and if so, on what dataset? What data augmentation techniques were applied? What measures were taken to prevent overfitting?

**Response**: Thank you for this comment. We have revised the manuscript to clarify the technical details of the AI model and the measures taken to prevent overfitting.

To explicitly indicate that our approach is based on transfer learning, we revised the terminology throughout the manuscript from “our model” to “our fine-tuned model” and clarified in the Methods as follows:

Lines: 190-191

*These models were pretrained on the ImageNet [16], a large-scale public dataset containing general images and their labels, and were subsequently fine-tuned using our dataset.*

Data augmentation was applied only to the training data and consisted of random horizontal flipping and contrast adjustment, which is written as follows:

Lines: 140-146

*Since the dataset was small, the data was augmented by randomly flipping and changing contrast. Augmentation was applied only for the training dataset.*

To mitigate overfitting, we adopted a small learning rate (1e-6) for stable fine-tuning, employed 5-fold cross-validation, and evaluated the final model using an independent hold-out test set. In addition, we added the learning curve of the fine-tuned InceptionV3 model (Figure 2), which suggests that no severe overfitting was observed.

Lines: 440-446

*Although the learning curves showed stable convergence without divergence of validation loss, a modest gap between training and validation loss remained, indicating a mild generalization gap rather than severe overfitting. Underfitting was unlikely, as both losses decreased steadily throughout training. This limitation is likely attributable to the relatively small, single-center dataset. Future work using larger, multi-institutional datasets, as well as additional regularization strategies and external validation, will be essential to further improve generalizability.*

5. More information needed about the clinical relevance and contextualise with the existing research out there. Did the authors consider the sensitivity/specifity for different disease stages? Did you account for age and ethnicity?

**Response:**
 Thank you for this comment. We have added a brief discussion to contextualize our findings with existing RP screening studies and to clarify the clinical relevance of our model on pages 25-26, lines356-376. Sensitivity and specificity were not stratified by disease stage, as detailed and standardized staging information was not consistently available across all patients in this retrospective dataset, and the primary objective of the study was to evaluate the feasibility of RP detection in a screening-oriented setting using fundus photographs alone. As discussed in the manuscript on page 34, lines 460-466, cases in which abnormalities are detectable on color fundus images are often considered to correspond to early to mid stages of the disease in routine clinical practice. We have clarified this point in the Discussion and explicitly noted stage-specific performance evaluation as an important direction for future work. With respect to age, demographic characteristics including age distribution were reported (Table 1), and the RP and non-RP groups showed comparable mean ages. However, no age-stratified subgroup analyses were performed, as the study was not powered to evaluate age-dependent effects on model performance. We acknowledge that age-related fundus changes could potentially influence predictions, and future studies with larger datasets will enable stratified or age-adjusted analyses. Regarding ethnicity, all images in this study were obtained from Japanese patients at a single tertiary referral center, resulting in a relatively homogeneous study population. Therefore, ethnicity-specific subgroup analyses were not feasible in the present study. These points have been acknowledged as limitations in the Discussion.

6. Major gaps in the literature review which makes it difficult to frame/contextualise this research. How does it compare to other studies? The following highly relevant studies should be cited:

<https://pubmed.ncbi.nlm.nih.gov/40567353/>

https://pubmed.ncbi.nlm.nih.gov/33879469/

https://pubmed.ncbi.nlm.nih.gov/31093368/

**Response**：

We thank the reviewer for this important comment. We agree that the initial literature review did not sufficiently contextualize our study. We have substantially revised the Introduction to include a more comprehensive discussion of prior work applying deep learning to inherited retinal diseases.

In particular, we now cite and discuss key studies on page 5, lines 74-83, including Fujinami-Yokokawa et al., who demonstrated gene prediction from fundus photography and fundus autofluorescence images, and Pontikos et al., who developed the Eye2Gene framework for large-scale, multimodal gene prediction in IRDs. We have clarified how our work differs from these studies by focusing specifically on RP classification using fundus photographs alone and emphasizing model interpretability in a retrospective, real-world clinical dataset. These revisions better position our study within the existing literature.
