# Supplementary material for: Fundus image analysis of retinitis pigmentosa using artificial intelligence
Source: PLoS One. 2026 Jul 24;21(7):e0354452. doi: 10.1371/journal.pone.0354452 (PMC13399350; doi:10.1371/journal.pone.0354452)
Supplement: Attachment — Submitted filename: renamed_c237e.docx [file pone.0354452.s003.docx]

**Response to reviewer comments**

Dear Dr. Vahid Mansouri

We would like to thank you for handling our manuscript and for providing us with the opportunity to revise it. We are also grateful to all reviewers for their careful reading of our work and for their constructive and insightful comments, which have helped us to substantially improve the quality, clarity, and rigor of the manuscript.

We have carefully considered each comment and have revised the manuscript accordingly. All changes have been made with the aim of addressing the reviewers’ concerns in a transparent manner.

1. If the authors have adequately addressed your comments raised in a previous round of review and you feel that this manuscript is now acceptable for publication, you may indicate that here to bypass the “Comments to the Author” section, enter your conflict of interest statement in the “Confidential to Editor” section, and submit your "Accept" recommendation.

Reviewer #1: All comments have been addressed

Reviewer #3: All comments have been addressed

Reviewer #4: (No Response)

2. Is the manuscript technically sound, and do the data support the conclusions?

The manuscript must describe a technically sound piece of scientific research with data that supports the conclusions. Experiments must have been conducted rigorously, with appropriate controls, replication, and sample sizes. The conclusions must be drawn appropriately based on the data presented.

Reviewer #1: Yes

Reviewer #3: Yes

Reviewer #4: Yes

3. Has the statistical analysis been performed appropriately and rigorously?

Reviewer #1: Yes

Reviewer #3: Yes

Reviewer #4: Yes

4. Have the authors made all data underlying the findings in their manuscript fully available?

The PLOS Data policy requires authors to make all data underlying the findings described in their manuscript fully available without restriction, with rare exception (please refer to the Data Availability Statement in the manuscript PDF file). The data should be provided as part of the manuscript or its supporting information, or deposited to a public repository. For example, in addition to summary statistics, the data points behind means, medians and variance measures should be available. If there are restrictions on publicly sharing data—e.g. participant privacy or use of data from a third party—those must be specified.

Reviewer #1: No

Response: We thank the reviewer for noting this important issue. The original dataset used in this study consists of color fundus photographs obtained from patient medical records. Because these medical images contain sensitive information and may carry a potential risk of personal identification, public deposition of the full dataset is restricted by patient privacy considerations and by the protocol approved by the Ethics Review Committee of Keio University School of Medicine. We have therefore revised the Data Availability Statement to clarify that the original image dataset cannot be made publicly available in a public repository. We have also clarified that de-identified data may be made available to qualified researchers upon reasonable request to the Ethics Review Committee of Keio University School of Medicine, subject to approval by the relevant institutional review board, compliance with institutional data protection policies, and completion of an appropriate data use agreement.

Reviewer #3: Yes

Reviewer #4: Yes

5. Is the manuscript presented in an intelligible fashion and written in standard English?

PLOS ONE does not copyedit accepted manuscripts, so the language in submitted articles must be clear, correct, and unambiguous. Any typographical or grammatical errors should be corrected at revision, so please note any specific errors here.

Reviewer #1: Yes

Reviewer #3: Yes

Reviewer #4: Yes

6. Review Comments to the Author

Please use the space provided to explain your answers to the questions above. You may also include additional comments for the author, including concerns about dual publication, research ethics, or publication ethics. (Please upload your review as an attachment if it exceeds 20,000 characters)

Reviewer #1: Paper is improved

Since the study is limited to a single-center dataset, future work should include multicenter external validation and more diverse ethnic populations to ensure broader generalizability.

Also, in future, you can evaluate the performance across different disease stages or subtypes to enhance its clinical utility.

Response: We thank the reviewer for the positive assessment and for these constructive suggestions. We agree that multicenter external validation in ethnically diverse cohorts is essential before clinical deployment. We have substantially revised and reorganized the “Limitations and future research” section to more explicitly acknowledge the absence of an independent external validation cohort and to clarify the need for future validation across diverse populations, imaging devices, disease stages, and genetic subtypes.

Reviewer #3: The authors have addressed most of my comments.

I suggest the following improvements to the writing.

Retinitis pigmentosa (RP) is one group of inherited retinal diseases that are caused by genetic defects that lead to progressive photoreceptor loss and eventual blindness → Retinitis pigmentosa (RP) is **a** group of inherited retinal diseases that are caused by genetic defects that lead to progressive photoreceptor loss and eventual blindness

Early diagnosis will be helpful for an effective management of the disease; however, many patients24stay unaware of early symptoms. → would be helpful

More recently, Pontikos et al. Developed Eye2gene → More recently, Pontikos et al. developed Eye2Gene

Several studies have investigated deep learning-based classification of RP using fundus images… → Studies that have investigated deep learning-based classification of RP using fundus images, include Guo et al…

Furthermore, we also performed gradient-weighted class activation mapping (Grad-CAM), which shows important pixels for prediction to mitigate the black-box nature of deep learning models. → This doesn’t belong in the introduction, I would remove this sentence.

a program for predicting RP using color fundus images is designed as shown in Figure 1 → an approach for training an AI to predict RP using color fundus images was designed as shown in Figure 1

Response: We thank the reviewer for carefully identifying these language and presentation issues. We have revised the manuscript accordingly. Specifically, we corrected the definition of retinitis pigmentosa, changed “will be helpful” to “would be helpful,” corrected the capitalization of Eye2Gene, revised the sentence introducing prior studies, removed the Grad-CAM sentence from the Introduction, and changed “a program for predicting RP” to “an approach for training an AI to predict RP.”

Reviewer #4: The current presentation of Figure 5 remains unconvincing. This finding may simply be incidental rather than disease-specific. In particular, the image shown on the right appears largely normal even from a clinical ophthalmologist’s perspective, and the manuscript does not provide sufficient evidence that the model is identifying a truly meaningful retinitis pigmentosa-related feature. The authors should therefore provide stronger supporting evidence for this example. At minimum, they should clarify the clinical diagnosis in greater detail and, if available, include corroborating multimodal evidence such as ultra-widefield imaging or OCT. This is especially important because the manuscript itself acknowledges that the study is based on a relatively small single-center dataset and does not include an external validation cohort.

More broadly, the lack of external validation remains a major weakness. For RP, public datasets do exist that could at least support preliminary external testing. For example, the JSIEC fundus dataset is publicly available as a 1,000-image subset spanning 39 categories and is presented as originating from multiple data sources, which could provide a more realistic test of generalizability than the current single-center data alone.

The literature review should also be strengthened. The manuscript would benefit from a broader discussion of recent AI efforts for rare retinal disease diagnosis, particularly studies that attempt to overcome the fundamental challenge of limited sample size in rare diseases. The recent paper on "macular hole detection and segmentation on fundus photography using large multimodal generative models for synthetic augmentation" is one relevant example, because it directly addresses the problem of scarce data through modern generative augmentation strategies rather than relying only on conventional transfer learning. In addition, recent reviews on AI in inherited retinal dystrophies emphasize that although promising results are emerging, robust external validation, multimodal integration, and clinically interpretable frameworks remain major unmet needs. These themes are highly relevant to the present manuscript and should be discussed in greater depth.

Response: We thank the reviewer for this important comment. We agree that the previous presentation of Figure 5 did not provide sufficient clinical context and could have been interpreted as incidental rather than disease-specific. We therefore performed an additional retrospective chart review of the two cases shown in Figure 5 and revised both the manuscript text and the figure legend. The chart review confirmed subtle peripheral degenerative changes and mild attenuation of retinal vessels in both cases. Although the peripheral findings were not readily apparent on initial inspection of the color fundus photographs, vascular attenuation was observed around the posterior pole. The Grad-CAM heatmaps highlighted regions around the optic disc and posterior pole, corresponding to the distribution of major retinal vascular structures. We have revised the manuscript to clarify that the model may have relied, at least in part, on secondary features such as vascular attenuation rather than overt peripheral pigmentation. We have also tempered the interpretation of Grad-CAM and now describe it as a qualitative visualization of model attention rather than definitive evidence that the model identified pathognomonic RP features. Multimodal images such as ultra-widefield imaging or OCT were not available for inclusion in the revised figure for these retrospective cases; therefore, we added the available clinical information from retrospective chart review and explicitly acknowledged the limitation of Grad-CAM interpretation.

We agree that the absence of an independent external validation cohort is a major limitation of the present study. We also agree that public datasets such as JSIEC may provide useful preliminary resources for external testing. However, rigorous external validation requires careful consideration of image acquisition conditions, diagnostic definitions, preprocessing pipelines, and disease-stage information. We therefore revised the “Limitations and future research” section to more explicitly acknowledge the lack of external validation and to clarify that future studies should perform multicenter validation across diverse populations, imaging devices, disease stages, and genetic subtypes. We also added that public fundus datasets may serve as useful preliminary resources, but clinically meaningful deployment will require larger, well-annotated, and demographically diverse cohorts. We revised the “Limitations and future research” section.

We agree that the literature review should better contextualize the present study within recent AI efforts for rare retinal disease diagnosis, particularly with respect to data scarcity, multimodal integration, external validation, and clinical interpretability. We therefore expanded the Discussion to include recent work on synthetic augmentation using large multimodal generative models for fundus photography, public fundus datasets that may support preliminary external testing, multimodal AI-based phenotyping of inherited retinal diseases, and recent reviews of AI applications in inherited retinal diseases. We also clarified that, although our study contributes to RP detection using color fundus photographs with internal cross-validation and Grad-CAM–based visualization, future work should include larger multicenter datasets, multimodal imaging approaches, and prospective external validation. We added the paragraph to the Discussion.

7. PLOS authors have the option to publish the peer review history of their article (what does this mean?). If published, this will include your full peer review and any attached files.

If you choose “no”, your identity will remain anonymous but your review may still be made public.

Do you want your identity to be public for this peer review? For information about this choice, including consent withdrawal, please see our Privacy Policy.

Reviewer #1:

Reviewer #3:

Reviewer #4:
