# Supplementary material for: Fundus image analysis of retinitis pigmentosa using artificial intelligence
Source: PLoS One. 2026 Jul 24;21(7):e0354452. doi: 10.1371/journal.pone.0354452 (PMC13399350; doi:10.1371/journal.pone.0354452)
Supplement: Attachment — Submitted filename: Response to Reviewers June.docx [file pone.0354452.s004.docx]

**Response to reviewer comments**

Dear Dr. Vahid Mansouri

We sincerely thank the Editor and Reviewers for their careful evaluation of our revised manuscript and for their constructive comments. We are pleased that the reviewers found that the previous comments had been satisfactorily addressed. In response to the remaining comments, we have revised the Abstract to clarify that both the retinitis pigmentosa and non-retinitis pigmentosa subjects were from Keio University Hospital and to include the number of subjects as well as the number of images. We have also clarified in the Methods section that data splitting was performed at the patient level to prevent potential data leakage from bilateral or multiple images obtained from the same patient.

In accordance with the journal requirements, we reviewed the reviewers’ comments and confirmed that no specific previously published works were recommended for citation in this round of review. We also reviewed the reference list to ensure that it is complete and accurate. No retracted articles were identified, and no changes were made to the reference list.

Our point-by-point responses to the reviewers’ comments are provided below.

Comments to the Author

1. If the authors have adequately addressed your comments raised in a previous round of review and you feel that this manuscript is now acceptable for publication, you may indicate that here to bypass the “Comments to the Author” section, enter your conflict of interest statement in the “Confidential to Editor” section, and submit your "Accept" recommendation.

Reviewer #1: All comments have been addressed

Reviewer #3: All comments have been addressed

Reviewer #4: All comments have been addressed

2. Is the manuscript technically sound, and do the data support the conclusions?

The manuscript must describe a technically sound piece of scientific research with data that supports the conclusions. Experiments must have been conducted rigorously, with appropriate controls, replication, and sample sizes. The conclusions must be drawn appropriately based on the data presented.

Reviewer #1: Yes

Reviewer #3: Yes

Reviewer #4: Yes

3. Has the statistical analysis been performed appropriately and rigorously?

Reviewer #1: N/A

Reviewer #3: Yes

Reviewer #4: Yes

4. Have the authors made all data underlying the findings in their manuscript fully available?

The PLOS Data policy requires authors to make all data underlying the findings described in their manuscript fully available without restriction, with rare exception (please refer to the Data Availability Statement in the manuscript PDF file). The data should be provided as part of the manuscript or its supporting information, or deposited to a public repository. For example, in addition to summary statistics, the data points behind means, medians and variance measures should be available. If there are restrictions on publicly sharing data—e.g. participant privacy or use of data from a third party—those must be specified.

Reviewer #1: Yes

Reviewer #3: Yes

Reviewer #4: Yes

5. Is the manuscript presented in an intelligible fashion and written in standard English?

PLOS ONE does not copyedit accepted manuscripts, so the language in submitted articles must be clear, correct, and unambiguous. Any typographical or grammatical errors should be corrected at revision, so please note any specific errors here.

Reviewer #1: Yes

Reviewer #3: Yes

Reviewer #4: Yes

6. Review Comments to the Author

Please use the space provided to explain your answers to the questions above. You may also include additional comments for the author, including concerns about dual publication, research ethics, or publication ethics. (Please upload your review as an attachment if it exceeds 20,000 characters)

Reviewer #1: Paper is improved Paper is in publishable quality.

Please proofread the paper for possible further improvements.

Response: We thank the reviewer for the positive evaluation. We have carefully proofread the manuscript and made minor edits to improve clarity and readability.

Reviewer #3: The authors have satisfactorily addressed all of my comments.

One minor suggestion for the abstract, is to make it clearer that both RP and non-RP patients are from the same source:

The dataset held 200 color fundus images of Japanese RP patients and 121 color fundus images from non-

RP subjects from Keio University Hospital, and multiple images were available for some patients.

->

The dataset comprised of Japanese subjects from Keio University Hospital, which included 200 color fundus images from RP patients and 121 color fundus images from non-RP subjects. Multiple images were available for some patients.

I would also suggest giving the number of subjects rather than images since there are repeat images per per patient (if word limit allows).

Response: We thank the reviewer for this helpful suggestion. We revised the Abstract to clarify that both RP and non-RP subjects were from Keio University Hospital and to provide the number of subjects as well as the number of images.

Reviewer #4: One methodological issue that requires clarification is the handling of bilateral or multiple images from the same patient. The manuscript notes that some patients contributed more than one image, but it remains unclear whether data splitting was performed at the patient level. Because retinitis pigmentosa is generally bilateral, fellow-eye images from the same patient are not statistically independent and may show similar disease-specific fundus features. Therefore, image-level random splitting could introduce data leakage if images from the same patient were distributed across training and test sets. The authors should clarify this point and confirm that all images from a given patient were kept within the same split, or otherwise provide a patient-level sensitivity analysis.

Response: We thank the reviewer for raising this important methodological point. We agree that bilateral or multiple images from the same patient are not statistically independent and that image-level splitting could introduce data leakage. In our analysis, data splitting was performed at the patient level. When multiple images, including bilateral images, were available from a single patient, all images from that patient were assigned exclusively to the same dataset partition. Therefore, no patient contributed images to more than one of the training, validation, or test datasets. We have clarified this point in the Methods section.

7. PLOS authors have the option to publish the peer review history of their article (what does this mean?). If published, this will include your full peer review and any attached files.

If you choose “no”, your identity will remain anonymous but your review may still be made public.

Do you want your identity to be public for this peer review? For information about this choice, including consent withdrawal, please see our Privacy Policy.

Reviewer #1: No

Reviewer #3: No

Reviewer #4: No
