# Supplementary material for: Fundus image analysis of retinitis pigmentosa using artificial intelligence
Source: PLoS One. 2026 Jul 24;21(7):e0354452. doi: 10.1371/journal.pone.0354452 (PMC13399350; doi:10.1371/journal.pone.0354452)
Supplement: Peer review history

# Peer review: Decision Letter 0

**DOI:** 10.1371/journal.pone.0354452.r001

**Authors:** Vahid Mansouri

26 Dec 2025

PONE-D-25-55224

Fundus Image Analysis of Retinitis Pigmentosa Using Artificial Intelligence

PLOS One

Dear Dr. Kurihara,

Thank you for submitting your manuscript to PLOS ONE. After careful consideration, we feel that it has merit but does not fully meet PLOS ONE’s publication criteria as it currently stands. Therefore, we invite you to submit a revised version of the manuscript that addresses the points raised during the review process.

Please submit your revised manuscript by Feb 09 2026 11:59PM. If you will need more time than this to complete your revisions, please reply to this message or contact the journal office at plosone@plos.org. When you're ready to submit your revision, log on to https://www.editorialmanager.com/pone/ and select the 'Submissions Needing Revision' folder to locate your manuscript file.

Please include the following items when submitting your revised manuscript:

- A letter that responds to each point raised by the academic editor and reviewer(s). You should upload this letter as a separate file labeled 'Response to Reviewers'.
- A marked-up copy of your manuscript that highlights changes made to the original version. You should upload this as a separate file labeled 'Revised Manuscript with Track Changes'.
- An unmarked version of your revised paper without tracked changes. You should upload this as a separate file labeled 'Manuscript'.

If you would like to make changes to your financial disclosure, please include your updated statement in your cover letter. Guidelines for resubmitting your figure files are available below the reviewer comments at the end of this letter.

If applicable, we recommend that you deposit your laboratory protocols in protocols.io to enhance the reproducibility of your results. Protocols.io assigns your protocol its own identifier (DOI) so that it can be cited independently in the future. For instructions see: https://journals.plos.org/plosone/s/submission-guidelines#loc-laboratory-protocols. Additionally, PLOS ONE offers an option for publishing peer-reviewed Lab Protocol articles, which describe protocols hosted on protocols.io. Read more information on sharing protocols at https://plos.org/protocols?utm_medium=editorial-email&utm_source=authorletters&utm_campaign=protocols.

We look forward to receiving your revised manuscript.

Kind regards,

Vahid Mansouri, M.D.

Academic Editor

PLOS One

Journal Requirements:

When submitting your revision, we need you to address these additional requirements.

1.Please ensure that your manuscript meets PLOS ONE's style requirements, including those for file naming. The PLOS ONE style templates can be found at

https://journals.plos.org/plosone/s/file?id=wjVg/PLOSOne_formatting_sample_main_body.pdf and

https://journals.plos.org/plosone/s/file?id=ba62/PLOSOne_formatting_sample_title_authors_affiliations.pdf

2. In the online submission form, you indicated that “The dataset is not publicly available due to its containing information that could compromise the privacy of research participants but are available from the corresponding author upon reasonable request.”

All PLOS journals now require all data underlying the findings described in their manuscript to be freely available to other researchers, either 1. In a public repository, 2. Within the manuscript itself, or 3. Uploaded as supplementary information.

This policy applies to all data except where public deposition would breach compliance with the protocol approved by your research ethics board. If your data cannot be made publicly available for ethical or legal reasons (e.g., public availability would compromise patient privacy), please explain your reasons on resubmission and your exemption request will be escalated for approval.

3. We note that you have indicated that there are restrictions to data sharing for this study. PLOS only allows data to be available upon request if there are legal or ethical restrictions on sharing data publicly. For more information on unacceptable data access restrictions, please see http://journals.plos.org/plosone/s/data-availability#loc-unacceptable-data-access-restrictions.

Before we proceed with your manuscript, please address the following prompts:

a) If there are ethical or legal restrictions on sharing a de-identified data set, please explain them in detail (e.g., data contain potentially identifying or sensitive patient information, data are owned by a third-party organization, etc.) and who has imposed them (e.g., a Research Ethics Committee or Institutional Review Board, etc.). Please also provide contact information for a data access committee, ethics committee, or other institutional body to which data requests may be sent.

b) If there are no restrictions, please upload the minimal anonymized data set necessary to replicate your study findings to a stable, public repository and provide us with the relevant URLs, DOIs, or accession numbers. For a list of recommended repositories, please see

https://journals.plos.org/plosone/s/recommended-repositories. You also have the option of uploading the data as Supporting Information files, but we would recommend depositing data directly to a data repository if possible.

We will update your Data Availability statement on your behalf to reflect the information you provide.

4. Please amend your Ethics Approval number to ensure that it matches in both the Manuscript and the Details page.

5. If the reviewer comments include a recommendation to cite specific previously published works, please review and evaluate these publications to determine whether they are relevant and should be cited. There is no requirement to cite these works unless the editor has indicated otherwise.

[Note: HTML markup is below. Please do not edit.]

Reviewer's Responses to Questions

**Comments to the Author**

1. Is the manuscript technically sound, and do the data support the conclusions?

The manuscript must describe a technically sound piece of scientific research with data that supports the conclusions. Experiments must have been conducted rigorously, with appropriate controls, replication, and sample sizes. The conclusions must be drawn appropriately based on the data presented.

Reviewer #1: Partly

Reviewer #2: Yes

Reviewer #3: Partly

**********

2. Has the statistical analysis been performed appropriately and rigorously?

Reviewer #1: N/A

Reviewer #2: Yes

Reviewer #3: Yes

**********

3. Have the authors made all data underlying the findings in their manuscript fully available?

The PLOS Data policy requires authors to make all data underlying the findings described in their manuscript fully available without restriction, with rare exception (please refer to the Data Availability Statement in the manuscript PDF file). The data should be provided as part of the manuscript or its supporting information, or deposited to a public repository. For example, in addition to summary statistics, the data points behind means, medians and variance measures should be available. If there are restrictions on publicly sharing data—e.g. participant privacy or use of data from a third party—those must be specified.

Reviewer #1: No

Reviewer #2: No

Reviewer #3: No

**********

4. Is the manuscript presented in an intelligible fashion and written in standard English?

PLOS ONE does not copyedit accepted manuscripts, so the language in submitted articles must be clear, correct, and unambiguous. Any typographical or grammatical errors should be corrected at revision, so please note any specific errors here.

Reviewer #1: Yes

Reviewer #2: Yes

Reviewer #3: Yes

**********

5. Review Comments to the Author

Please use the space provided to explain your answers to the questions above. You may also include additional comments for the author, including concerns about dual publication, research ethics, or publication ethics. (Please upload your review as an attachment if it exceeds 20,000 characters)

Reviewer #1: The study presents an approach for Retinitis Pigmentosa diagnosis from fundus images.

The following improvements are suggested.

1. It would be better to highlight the main research questions in the introduction section, and describe in the Discussion section, how you fulfilled those in the study, by referring to the applied methods and the obtained results.

2. Introduction - state the gaps in the latest literature including https://doi.org/10.1016/j.imu.2025.101644 and how the proposed research aims to address them.

3. Provide a rationale for the chosen hyperparameters: learning rate (0.000001), number of epochs (200), and the use of the Adam optimizer. Were other values tested, and what was the impact on performance?

4. Proofread for typographic errors. Eg. Line 152 mentions that Inception V3 attained the best performance in accuracy in Table 1 , but Table 1 is the Patient demographic characteristics. The performance metrics are in Table 2. Please correct the in-text reference from Table 1 to Table 2 for the cross-validation results.

5. The Abstract states the dataset contained 200 RP images and 121 non-RP images. The Results section mentions 200 RP images from 107 patients and 121 non-RP images from 94 patients. It should be confirmed that the study uses 200 RP images and 121 Non-RP images (Total 321 images) for the classification task, and that some patients contributed multiple images.

6. Describe the implementation details such as used libraries and the hardware specification (CPU/ GPU)

7. Include more evaluation metrics such as confusion matrix and the learning curves (training and validation/ accuracy and loss)

8. Discuss the issues and limitations (such as overfitting/ underfitting) in the training model and how you can address those.

9. For all the results figures and tables, clearly explain the observations and the decisions that can be taken by those observations. Eg. Figure 3 -, clarify what the Grad-CAM heatmap is highlighting. Since the image is correctly classified as Non-RP, the heatmap indicates the regions that most contributed to the model's decision not to predict RP. Discuss whether these "important pixels" align with expected normal fundus features, or if they point to regions the model actively avoids focusing on.

10. Include a comparison table in the discussion, stating the new features and results of the proposed model, in comparison to the latest such studies. By that, justify your novel contribution.

11. Discuss the future work and possibility of deploying the proposed model as a real world application in clinical settings such as in https://doi.org/10.1109/ACCESS.2024.3359698. Because most of the AI based models are not implemented for actual practice.

12. What is the possibility of validating this approach with medical experts.

Reviewer #2: This study explored the potential of deep learning for detecting retinitis pigmentosa (RP) from color fundus images. Using transfer learning, three pre-trained models were fine-tuned on a dataset of 321 fundus images from Keio University Hospital. Among these, InceptionV3 achieved the highest accuracy (96.97%, matching the average diagnostic performance of ophthalmologists. Gradient-weighted Class Activation Mapping (Grad-CAM) confirmed the model’s predominant region of interest. However, several limitations need to be addressed to enhance its scientific rigor and clinical applicability.

1.The manuscript stated the dataset was separated into training and test sets but failed to specify the ratio, rationale, and method for this split. Furthermore, the exact size of the training set after augmentation was not provided.

Authors must explicitly detail the exact proportion of training and test sets, including the augmentation samples.

2.The manuscript only displayed Grad-CAM heatmaps for misdiagnosed samples, failing to contrast the regions of focus between correctly and incorrectly diagnosed cases.

Authors should provide visual evidence demonstrating that heatmaps of true positive samples indeed concentrate on the peripheral retina.

3.There was no statistical comparison, like confidence intervals and p-values, to quantify the differences between models and support the claim that the model's performance was comparable to ophthalmologists.

4.The patient demographic description was overly simplistic. For an inherited disease like RP, the family history is critical. Its absence from the dataset description raised questions about the clinical representativeness of the RP patients.

Authors should supplement Table 1 to include the proportion of patients with family history of RP. If this information is unavailable from the retrospective records, it should be explicitly stated and discussed in the manuscript.

5.The manuscript noted the use of cross-validation as a distinction from some prior work but failed to explain why this was methodologically critical. The purpose and advantages of CV was not fully articulated.

In the Discussion, authors must explicitly state the role of k-fold cross-validation.

6.Description of the data in Table 3 in line 158 was inconsistent with the actual table content.

Furthermore, several inconsistencies and errors reduce the manuscript's polish.

1.There are textural errors in line 24, line 25, line 229, line 249.

2.The legend for Table 2 was incorrectly positioned in line 161.

Reviewer #3: This study by Ubukata and colleagues, presents an AI-based approach for analyzing fundus images of patients with Retinitis Pigmentosa (RP). The authors developed and evaluated deep learning models to assess disease characteristics and severity from fundus photographs, aiming to provide an automated tool for clinical assessment.

The topic is highly relevant as automated analysis of RP fundus images addresses a real need for objective, quantifiable disease assessment methods. If validated, such tools could assist in monitoring disease progression and treatment response.

I would like however see the following issues addressed to strengthen the manuscript:

1. The sample size and diversity of the training dataset need clarification. The author should specify many patients and images were included. Whether images from multiple centers included to ensure generalizability? How was the dataset split for training, validation, and testing? Were external validation cohorts used?

2. The establishment of ground truth labels is critical but insufficiently described. Who determined the ground truth labels for RP (single expert, consensus panel)? Was there a genetic diagnosis to confirm RP? Did you consider different subtypes of RP?

3. More details about the imaging needed. What retina scanner was used to acquire the images, where the pupils dilated on acquisition? Bad quality images are mentioned. What was the criteria to exclude bad quality images? Were any other preprocessing step performs like normalisation or cropping on the images?

4. More technical details about the AI model needed. Were pre-trained models like a foundation model used, and if so, on what dataset? What data augmentation techniques were applied? What measures were taken to prevent overfitting?

5. More information needed about the clinical relevance and contextualise with the existing research out there. Did the authors consider the sensitivity/specifity for different disease stages? Did you account for age and ethnicity?

6. Major gaps in the literature review which makes it difficult to frame/contextualise this research. How does it compare to other studies? The following highly relevant studies should be cited:

https://pubmed.ncbi.nlm.nih.gov/40567353/

https://pubmed.ncbi.nlm.nih.gov/31093368/

https://pubmed.ncbi.nlm.nih.gov/33879469/

**********

6. PLOS authors have the option to publish the peer review history of their article (what does this mean?). If published, this will include your full peer review and any attached files.

If you choose “no”, your identity will remain anonymous but your review may still be made public.

**Do you want your identity to be public for this peer review?**  For information about this choice, including consent withdrawal, please see our Privacy Policy.

Reviewer #1: No

Reviewer #2: No

Reviewer #3: No

**********

[NOTE: If reviewer comments were submitted as an attachment file, they will be attached to this email and accessible via the submission site. Please log into your account, locate the manuscript record, and check for the action link "View Attachments". If this link does not appear, there are no attachment files.]

To ensure your figures meet our technical requirements, please review our figure guidelines: https://journals.plos.org/plosone/s/figures

You may also use PLOS’s free figure tool, NAAS, to help you prepare publication quality figures: https://journals.plos.org/plosone/s/figures#loc-tools-for-figure-preparation.

NAAS will assess whether your figures meet our technical requirements by comparing each figure against our figure specifications.

# Author comment: Author response to Decision Letter 1

**DOI:** 10.1371/journal.pone.0354452.r002

20 Feb 2026

Response to reviewer comments

Dear Dr. Vahid Mansouri

We would like to thank you for handling our manuscript and for providing us with the opportunity to revise it. We are also grateful to all reviewers for their careful reading of our work and for their constructive and insightful comments, which have helped us to substantially improve the quality, clarity, and rigor of the manuscript.

We have carefully considered each comment and have revised the manuscript accordingly. All changes have been made with the aim of addressing the reviewers’ concerns in a transparent manner.

Below, we provide a point-by-point response to each comment raised by the reviewers. Reviewer comments are reproduced with a colored background for clarity, followed by our responses. Where relevant, revised or newly added text from the main manuscript is shown in italics.

Journal Requirements

1. Please ensure that your manuscript meets PLOS ONE's style requirements, including those for file naming. The PLOS ONE style templates can be found at

https://journals.plos.org/plosone/s/file?id=wjVg/PLOSOne_formatting_sample_main_body.pdf

and

https://journals.plos.org/plosone/s/file?id=ba62/PLOSOne_formatting_sample_title_authors_affiliations.pdf

2. In the online submission form, you indicated that “The dataset is not publicly available due to its containing information that could compromise the privacy of research participants but are available from the corresponding author upon reasonable request.”

All PLOS journals now require all data underlying the findings described in their manuscript to be freely available to other researchers, either

1. In a public repository,

2. Within the manuscript itself, or

3. Uploaded as supplementary information.

This policy applies to all data except where public deposition would breach compliance with the protocol approved by your research ethics board.

If your data cannot be made publicly available for ethical or legal reasons (e.g., public availability would compromise patient privacy), please explain your reasons on resubmission and your exemption request will be escalated for approval.

Response:

Thank you for this important clarification regarding the PLOS data availability policy. The primary data underlying this study consist of retinal fundus images obtained from human participants. Due to the unique and potentially identifiable nature of retinal vascular patterns, public deposition of these images could compromise patient privacy, even after de-identification. Therefore, public sharing of the image dataset is not permitted under the protocol approved by the Ethics Committee of Keio University School of Medicine and is restricted for ethical reasons. We respectfully request an exemption from public data deposition for the image dataset based on these ethical restrictions. To ensure reproducibility of our findings, we confirm that all information necessary to replicate the analyses, including the software libraries, model architectures, training procedures, cross-validation strategy, and hyperparameters, has been fully described in the Methods section of the manuscript. In addition, the summary results and evaluation metrics are provided within the manuscript. The Data Availability Statement has been revised accordingly to reflect these points.

3. We note that you have indicated that there are restrictions to data sharing for this study. PLOS only allows data to be available upon request if there are legal or ethical restrictions on sharing data publicly.

For more information on unacceptable data access restrictions, please see

http://journals.plos.org/plosone/s/data-availability#loc-unacceptable-data-access-restrictions

Before we proceed with your manuscript, please address the following prompts:

a) If there are ethical or legal restrictions on sharing a de-identified data set, please explain them in detail (e.g., data contain potentially identifying or sensitive patient information, data are owned by a third-party organization, etc.) and who has imposed them (e.g., a Research Ethics Committee or Institutional Review Board, etc.). Please also provide contact information for a data access committee, ethics committee, or other institutional body to which data requests may be sent.

b) If there are no restrictions, please upload the minimal anonymized data set necessary to replicate your study findings to a stable, public repository and provide us with the relevant URLs, DOIs, or accession numbers.

For a list of recommended repositories, please see

https://journals.plos.org/plosone/s/recommended-repositories

You also have the option of uploading the data as Supporting Information files, but we would recommend depositing data directly to a data repository if possible.

We will update your Data Availability statement on your behalf to reflect the information you provide.

Response:

Thank you for this comment. The dataset consists of retinal fundus images from human subjects, which may retain potentially identifying features even after de-identification. Therefore, public data sharing is ethically restricted to protect patient privacy. In addition, explicit informed consent for public data sharing was not obtained from the participants at the time of data collection. Therefore, unrestricted public release of the raw image data is not permitted. The Data Availability Statement has been revised accordingly. Requests for data access may be directed to the Ethics Committee of Keio University School of Medicine or the corresponding author.

4. Please amend your Ethics Approval number to ensure that it matches in both the Manuscript and the Details page.

Response:

Thank you for pointing this out. We carefully re-checked the Ethics Approval number in both the manuscript and the online submission details page and confirm that the approval number is now consistent in both locations.

5. If the reviewer comments include a recommendation to cite specific previously published works, please review and evaluate these publications to determine whether they are relevant and should be cited. There is no requirement to cite these works unless the editor has indicated otherwise.

Response:

We carefully reviewed the previously published works recommended by the reviewer and evaluated their relevance to the present study. Based on this assessment, we have cited the publications that were directly relevant and appropriate to the scope of our research. The manuscript has been revised accordingly.

Reviewer #1

1. It would be better to highlight the main research questions in the introduction section, and describe in the Discussion section, how you fulfilled those in the study, by referring to the applied methods and the obtained results.

Response:

Thank you for this helpful suggestion. We have revised the Introduction section to more clearly highlight the main research questions of the study on page 7, lines 114-121. In addition, we have expanded the Discussion section to explicitly address how these research questions were fulfilled, with reference to the applied methods and the obtained results on page 28, lines 411-414. These revisions were made to improve the clarity and coherence of the manuscript.

2. Introduction - state the gaps in the latest literature including https://doi.org/10.1016/j.imu.2025.101644 and how the proposed research aims to address them.

Response:

We thank the reviewer for this helpful suggestion. We have revised the Introduction to reference a recent systematic review on deep-learning–based glaucoma identification and to highlight the gap between the well-developed glaucoma literature and the relatively limited, less explainable deep-learning research in retinitis pigmentosa on pages 4-5, lines 64-73. We also clarified how our proposed framework addresses this gap by emphasizing both diagnostic performance and Grad-CAM–based interpretability.

3. Provide a rationale for the chosen hyperparameters: learning rate (0.000001), number of epochs (200), and the use of the Adam optimizer. Were other values tested, and what was the impact on performance?

Response:

We thank the reviewer for this helpful comment. We have clarified the rationale for the selected hyperparameters in the Methods section on pages 12-13, lines 193-202, including the learning rate, number of epochs, and the use of the Adam optimizer. Other learning rates were explored in preliminary experiments, and the selection was based on observed training stability and convergence behavior.

The following text has been added to the manuscript:

Adam optimizer was used for all models, as it is widely adopted in deep learning–based image classification tasks and provides stable and efficient optimization through adaptive learning rates and momentum-based updates, particularly in transfer learning settings. The learning rate was set to 1e-6, which was selected based on preliminary exploratory experiments. Learning rates at different orders of magnitude were tested during initial runs, and 1e-6 showed the most stable convergence behavior, whereas higher values occasionally led to unstable training and lower values resulted in slower convergence. The number of training epochs was set to 200 to ensure sufficient convergence when using this relatively small learning rate. Cross-entropy loss was used as the loss function.

4. Proofread for typographic errors. Eg. Line 152 mentions that Inception V3 attained the best performance in accuracy in Table 1, but Table 1 is the Patient demographic characteristics. The performance metrics are in Table 2. Please correct the in-text reference from Table 1 to Table 2 for the cross-validation results.

Response:

Thank you for pointing out this typographical error. We have carefully proofread the manuscript and corrected the in-text reference in Line 258. The performance metrics of the models, including the accuracy achieved by Inception V3, are now correctly referenced to Table 2, which presents the cross-validation results, instead of Table 1 (patient demographic characteristics). The manuscript has been revised accordingly.

5. The Abstract states the dataset contained 200 RP images and 121 non-RP images. The Results section mentions 200 RP images from 107 patients and 121 non-RP images from 94 patients. It should be confirmed that the study uses 200 RP images and 121 Non-RP images (Total 321 images) for the classification task, and that some patients contributed multiple images.

Response:

Thank you for this important comment. We confirm that the classification task consistently used 200 RP images and 121 non-RP images (total 321 images) throughout the study. As noted in the Results section, these images were obtained from 107 RP patients and 94 non-RP patients, and some patients contributed images from both eyes, resulting in multiple images per patient.

To avoid confusion, we have revised the Results section (page 15, lines 236-239) to explicitly state that the dataset includes bilateral eye images from certain patients, while the total number of images used for classification remains unchanged.

6. Describe the implementation details such as used libraries and the hardware specification (CPU/ GPU)

Response:

We thank the reviewer for this helpful suggestion. In response, we have updated the Materials and Methods section (page 13, lines 213-216) to explicitly describe the implementation environment. The revised text now specifies the software libraries used for preprocessing, model implementation, and visualization, as well as the hardware configuration used for training and inference as follows:

All models were implemented using PyTorch. Data preprocessing was performed using OpenCV, and model performance was visualized using Matplotlib. Model training and inference were conducted on an Amazon Web Services (AWS) p3 instance equipped with an NVIDIA V100 Tensor Core GPU.

7. Include more evaluation metrics such as confusion matrix and the learning curves (training and validation/ accuracy and loss)

8. Discuss the issues and limitations (such as overfitting/ underfitting) in the training model and how you can address those.

Response: We thank the reviewer for these constructive comments. In response, we have added the learning curves of the fine-tuned InceptionV3 model to the main text as Figure 2, showing the mean training and validation losses across five-fold cross-validation. Confusion matrix is shown as Table 3.

We have also expanded the Discussion section (page 30, lines 440-446) to explicitly address potential overfitting, its likely cause, and potential solution, as follows:

Although the learning curves showed stable convergence without divergence of validation loss, a modest gap between training and validation loss remained, indicating a mild generalization gap rather than severe overfitting. Underfitting was unlikely, as both losses decreased steadily throughout training. This limitation is likely attributable to the relatively small, single-center dataset. Future work using larger, multi-institutional datasets, as well as additional regularization strategies and external validation, will be essential to further improve generalizability.

9. For all the results figures and tables, clearly explain the observations and the decisions that can be taken by those observations. Eg. Figure 3 -, clarify what the Grad-CAM heatmap is highlighting. Since the image is correctly classified as Non-RP, the heatmap indicates the regions that most contributed to the model's decision not to predict RP. Discuss whether these "important pixels" align with expected normal fundus features, or if they point to regions the model actively avoids focusing on.

Response:

We thank the reviewer for this valuable comment. We have revised the Discussion section to explicitly describe what the Grad-CAM heatmaps highlight and how these regions contribute to the model’s decisions on pages 26-27, lines 377-402. We now clarify that in both true positive and true negative cases the model focuses on peripheral regions, reflecting evaluation of the presence or absence of typical peripheral degenerative changes. We also explain that false positive cases arise from incorrect attention to central macular patterns. These revisions clarify the interpretability of Figure 4 and the clinical relevance of the highlighted regions.

10. Include a comparison table in the discussion, stating the new features and results of the proposed model, in comparison to the latest such studies. By that, justify your novel contribution.

Response:

We thank the reviewer for this valuable suggestion. To clearly highlight the novelty of our work, we have added a comparison table (Table 5) in the Discussion section, summarizing recent RP classification studies in terms of model architecture, validation strategy, performance, and interpretability. This table demonstrates that our proposed model uniquely combines rigorous cross-validation with Grad-CAM–based explainability while achieving competitive or superior diagnostic performance, thereby clarifying our novel contribution.

11. Discuss the future work and possibility of deploying the proposed model as a real world application in clinical settings such as in https://doi.org/10.1109/ACCESS.2024.3359698. Because most of the AI based models are not implemented for actual practice.

Response:

Thank you for this important suggestion. We have added a new paragraph in the Discussion section addressing the future work and real-world deployment potential of our model, in reference to recent clinically oriented studies. We clarify that our model is intended as a decision-support tool for RP screening and triage rather than a standalone diagnostic system. We also discuss key requirements for clinical translation, including multicenter external validation, robustness across imaging devices, workflow integration, and prospective evaluation. In addition, we highlight the role of Grad-CAM–based explainability in improving clinical trust and adoption.

Added text in the Discussion

Lines:100-113

Beyond interpretability, practical deployment in real-world clinical settings remains a critical challenge for AI-based fundus image analysis. Recent clinically oriented studies have demonstrated the feasibility of deploying

Attachment Submitted filename: response_to_reviewers unmarked.docx

# Peer review: Decision Letter 1

**DOI:** 10.1371/journal.pone.0354452.r003

**Authors:** Vahid Mansouri

13 Apr 2026

PONE-D-25-55224R1

Fundus Image Analysis of Retinitis Pigmentosa Using Artificial Intelligence

PLOS One

Dear Dr. Kurihara,

Thank you for submitting your manuscript to PLOS ONE. After careful consideration, we feel that it has merit but does not fully meet PLOS ONE’s publication criteria as it currently stands. Therefore, we invite you to submit a revised version of the manuscript that addresses the points raised during the review process.

Please submit your revised manuscript by May 28 2026 11:59PM. If you will need more time than this to complete your revisions, please reply to this message or contact the journal office at plosone@plos.org. When you're ready to submit your revision, log on to https://www.editorialmanager.com/pone/ and select the 'Submissions Needing Revision' folder to locate your manuscript file.

Please include the following items when submitting your revised manuscript:

- A letter that responds to each point raised by the academic editor and reviewer(s). You should upload this letter as a separate file labeled 'Response to Reviewers'.
- A marked-up copy of your manuscript that highlights changes made to the original version. You should upload this as a separate file labeled 'Revised Manuscript with Track Changes'.
- An unmarked version of your revised paper without tracked changes. You should upload this as a separate file labeled 'Manuscript'.

If you would like to make changes to your financial disclosure, please include your updated statement in your cover letter. Guidelines for resubmitting your figure files are available below the reviewer comments at the end of this letter.

If applicable, we recommend that you deposit your laboratory protocols in protocols.io to enhance the reproducibility of your results. Protocols.io assigns your protocol its own identifier (DOI) so that it can be cited independently in the future. For instructions see: https://journals.plos.org/plosone/s/submission-guidelines#loc-laboratory-protocols. Additionally, PLOS ONE offers an option for publishing peer-reviewed Lab Protocol articles, which describe protocols hosted on protocols.io. Read more information on sharing protocols at https://plos.org/protocols?utm_medium=editorial-email&utm_source=authorletters&utm_campaign=protocols.

As the corresponding author, your ORCID iD is verified in the submission system and will appear in the published article. PLOS supports the use of ORCID, and we encourage all coauthors to register for an ORCID iD and use it as well. Please encourage your coauthors to verify their ORCID iD within the submission system before final acceptance, as unverified ORCID iDs will not appear in the published article. *Only*  the individual author can complete the verification step; PLOS staff *cannot*  verify ORCID iDs on behalf of authors.

We look forward to receiving your revised manuscript.

Kind regards,

Vahid Mansouri, M.D.

Academic Editor

PLOS One

Journal Requirements:

1. If the reviewer comments include a recommendation to cite specific previously published works, please review and evaluate these publications to determine whether they are relevant and should be cited. There is no requirement to cite these works unless the editor has indicated otherwise.

2. Please review your reference list to ensure that it is complete and correct. If you have cited papers that have been retracted, please include the rationale for doing so in the manuscript text, or remove these references and replace them with relevant current references. Any changes to the reference list should be mentioned in the rebuttal letter that accompanies your revised manuscript. If you need to cite a retracted article, indicate the article’s retracted status in the References list and also include a citation and full reference for the retraction notice.

[Note: HTML markup is below. Please do not edit.]

Reviewers' comments:

Reviewer's Responses to Questions

**Comments to the Author**

1. If the authors have adequately addressed your comments raised in a previous round of review and you feel that this manuscript is now acceptable for publication, you may indicate that here to bypass the “Comments to the Author” section, enter your conflict of interest statement in the “Confidential to Editor” section, and submit your "Accept" recommendation.

Reviewer #1: All comments have been addressed

Reviewer #3: All comments have been addressed

Reviewer #4: (No Response)

**********

2. Is the manuscript technically sound, and do the data support the conclusions?

The manuscript must describe a technically sound piece of scientific research with data that supports the conclusions. Experiments must have been conducted rigorously, with appropriate controls, replication, and sample sizes. The conclusions must be drawn appropriately based on the data presented.

Reviewer #1: Yes

Reviewer #3: Yes

Reviewer #4: Yes

**********

3. Has the statistical analysis been performed appropriately and rigorously?

Reviewer #1: Yes

Reviewer #3: Yes

Reviewer #4: Yes

**********

4. Have the authors made all data underlying the findings in their manuscript fully available?

The PLOS Data policy requires authors to make all data underlying the findings described in their manuscript fully available without restriction, with rare exception (please refer to the Data Availability Statement in the manuscript PDF file). The data should be provided as part of the manuscript or its supporting information, or deposited to a public repository. For example, in addition to summary statistics, the data points behind means, medians and variance measures should be available. If there are restrictions on publicly sharing data—e.g. participant privacy or use of data from a third party—those must be specified.

Reviewer #1: No

Reviewer #3: Yes

Reviewer #4: Yes

**********

5. Is the manuscript presented in an intelligible fashion and written in standard English?

PLOS ONE does not copyedit accepted manuscripts, so the language in submitted articles must be clear, correct, and unambiguous. Any typographical or grammatical errors should be corrected at revision, so please note any specific errors here.

Reviewer #1: Yes

Reviewer #3: Yes

Reviewer #4: Yes

**********

6. Review Comments to the Author

Please use the space provided to explain your answers to the questions above. You may also include additional comments for the author, including concerns about dual publication, research ethics, or publication ethics. (Please upload your review as an attachment if it exceeds 20,000 characters)

Reviewer #1: Paper is improved

Since the study is limited to a single-center dataset, future work should include multicenter external validation and more diverse ethnic populations to ensure broader generalizability.

Also, in future, you can evaluate the performance across different disease stages or subtypes to enhance its clinical utility.

Reviewer #3: The authors have addressed most of my comments.

I suggest the following improvements to the writing.

Retinitis pigmentosa (RP) is one group of inherited retinal diseases that are caused by genetic defects that lead to progressive photoreceptor loss and eventual blindness → Retinitis pigmentosa (RP) is **a** group of inherited retinal diseases that are caused by genetic defects that lead to progressive photoreceptor loss and eventual blindness

Early diagnosis will be helpful for an effective management of the disease; however, many patients24stay unaware of early symptoms. → would be helpful

More recently, Pontikos et al. Developed Eye2gene → More recently, Pontikos et al. developed Eye2Gene

Several studies have investigated deep learning-based classification of RP using fundus images… → Studies that have investigated deep learning-based classification of RP using fundus images, include Guo et al…

Furthermore, we also performed gradient-weighted class activation mapping (Grad-CAM), which shows important pixels for prediction to mitigate the black-box nature of deep learning models. → This doesn’t belong in the introduction, I would remove this sentence.

a program for predicting RP using color fundus images is designed as shown in Figure 1 → an approach for training an AI to predict RP using color fundus images was designed as shown in Figure 1

Reviewer #4: The current presentation of Figure 5 remains unconvincing. This finding may simply be incidental rather than disease-specific. In particular, the image shown on the right appears largely normal even from a clinical ophthalmologist’s perspective, and the manuscript does not provide sufficient evidence that the model is identifying a truly meaningful retinitis pigmentosa-related feature. The authors should therefore provide stronger supporting evidence for this example. At minimum, they should clarify the clinical diagnosis in greater detail and, if available, include corroborating multimodal evidence such as ultra-widefield imaging or OCT. This is especially important because the manuscript itself acknowledges that the study is based on a relatively small single-center dataset and does not include an external validation cohort.

More broadly, the lack of external validation remains a major weakness. For RP, public datasets do exist that could at least support preliminary external testing. For example, the JSIEC fundus dataset is publicly available as a 1,000-image subset spanning 39 categories and is presented as originating from multiple data sources, which could provide a more realistic test of generalizability than the current single-center data alone.

The literature review should also be strengthened. The manuscript would benefit from a broader discussion of recent AI efforts for rare retinal disease diagnosis, particularly studies that attempt to overcome the fundamental challenge of limited sample size in rare diseases. The recent paper on "macular hole detection and segmentation on fundus photography using large multimodal generative models for synthetic augmentation" is one relevant example, because it directly addresses the problem of scarce data through modern generative augmentation strategies rather than relying only on conventional transfer learning. In addition, recent reviews on AI in inherited retinal dystrophies emphasize that although promising results are emerging, robust external validation, multimodal integration, and clinically interpretable frameworks remain major unmet needs. These themes are highly relevant to the present manuscript and should be discussed in greater depth.

**********

7. PLOS authors have the option to publish the peer review history of their article (what does this mean?). If published, this will include your full peer review and any attached files.

If you choose “no”, your identity will remain anonymous but your review may still be made public.

**Do you want your identity to be public for this peer review?**  For information about this choice, including consent withdrawal, please see our Privacy Policy.

Reviewer #1:

Reviewer #3:

Reviewer #4:

**********

[NOTE: If reviewer comments were submitted as an attachment file, they will be attached to this email and accessible via the submission site. Please log into your account, locate the manuscript record, and check for the action link "View Attachments". If this link does not appear, there are no attachment files.]

To ensure your figures meet our technical requirements, please review our figure guidelines: https://journals.plos.org/plosone/s/figures

You may also use PLOS’s free figure tool, NAAS, to help you prepare publication quality figures: https://journals.plos.org/plosone/s/figures#loc-tools-for-figure-preparation.

NAAS will assess whether your figures meet our technical requirements by comparing each figure against our figure specifications.

# Author comment: Author response to Decision Letter 2

**DOI:** 10.1371/journal.pone.0354452.r004

31 May 2026

We sincerely thank the editor and reviewers for their careful evaluation of our revised manuscript. We have revised the manuscript in response to all reviewer comments and have provided detailed point-by-point responses in the uploaded Response to Reviewers document.

In this revision, we corrected the language and presentation issues raised by Reviewer #3. We also substantially revised and reorganized the “Limitations and future research” section to more explicitly acknowledge the absence of an independent external validation cohort and to clarify the need for future multicenter validation across diverse populations, imaging devices, disease stages, and genetic subtypes.

In response to Reviewer #4, we revised the description and legend of Figure 5 by adding clinical information obtained from retrospective chart review and by tempering the interpretation of Grad-CAM. We also expanded the Discussion to include recent literature on AI applications in rare and inherited retinal diseases, including data scarcity, synthetic augmentation, public fundus datasets, multimodal integration, external validation, and clinical interpretability.

A detailed point-by-point response to each reviewer comment is provided in the uploaded Response to Reviewers file.

Attachment Submitted filename: renamed_c237e.docx

# Peer review: Decision Letter 2

**DOI:** 10.1371/journal.pone.0354452.r005

**Authors:** Vahid Mansouri

14 Jun 2026

PONE-D-25-55224R2

Fundus Image Analysis of Retinitis Pigmentosa Using Artificial Intelligence

PLOS One

Dear Dr. Kurihara,

Thank you for submitting your manuscript to PLOS ONE. After careful consideration, we feel that it has merit but does not fully meet PLOS ONE’s publication criteria as it currently stands. Therefore, we invite you to submit a revised version of the manuscript that addresses the points raised during the review process.

Please submit your revised manuscript by Jul 29 2026 11:59PM. If you will need more time than this to complete your revisions, please reply to this message or contact the journal office at plosone@plos.org. When you're ready to submit your revision, log on to https://www.editorialmanager.com/pone/ and select the 'Submissions Needing Revision' folder to locate your manuscript file.

Please include the following items when submitting your revised manuscript:

- A letter that responds to each point raised by the academic editor and reviewer(s). You should upload this letter as a separate file labeled 'Response to Reviewers'.
- A marked-up copy of your manuscript that highlights changes made to the original version. You should upload this as a separate file labeled 'Revised Manuscript with Track Changes'.
- An unmarked version of your revised paper without tracked changes. You should upload this as a separate file labeled 'Manuscript'.

If you would like to make changes to your financial disclosure, please include your updated statement in your cover letter. Guidelines for resubmitting your figure files are available below the reviewer comments at the end of this letter.

If applicable, we recommend that you deposit your laboratory protocols in protocols.io to enhance the reproducibility of your results. Protocols.io assigns your protocol its own identifier (DOI) so that it can be cited independently in the future. For instructions see: https://journals.plos.org/plosone/s/submission-guidelines#loc-laboratory-protocols. Additionally, PLOS ONE offers an option for publishing peer-reviewed Lab Protocol articles, which describe protocols hosted on protocols.io. Read more information on sharing protocols at https://plos.org/protocols?utm_medium=editorial-email&utm_source=authorletters&utm_campaign=protocols.

As the corresponding author, your ORCID iD is verified in the submission system and will appear in the published article. PLOS supports the use of ORCID, and we encourage all coauthors to register for an ORCID iD and use it as well. Please encourage your coauthors to verify their ORCID iD within the submission system before final acceptance, as unverified ORCID iDs will not appear in the published article. *Only*  the individual author can complete the verification step; PLOS staff *cannot*  verify ORCID iDs on behalf of authors.

We look forward to receiving your revised manuscript.

Kind regards,

Vahid Mansouri, M.D.

Academic Editor

PLOS One

Journal Requirements:

If the reviewer comments include a recommendation to cite specific previously published works, please review and evaluate these publications to determine whether they are relevant and should be cited. There is no requirement to cite these works unless the editor has indicated otherwise.

Please review your reference list to ensure that it is complete and correct. If you have cited papers that have been retracted, please include the rationale for doing so in the manuscript text, or remove these references and replace them with relevant current references. Any changes to the reference list should be mentioned in the rebuttal letter that accompanies your revised manuscript. If you need to cite a retracted article, indicate the article’s retracted status in the References list and also include a citation and full reference for the retraction notice.

[Note: HTML markup is below. Please do not edit.]

Reviewers' comments:

Reviewer's Responses to Questions

**Comments to the Author**

1. If the authors have adequately addressed your comments raised in a previous round of review and you feel that this manuscript is now acceptable for publication, you may indicate that here to bypass the “Comments to the Author” section, enter your conflict of interest statement in the “Confidential to Editor” section, and submit your "Accept" recommendation.

Reviewer #1: All comments have been addressed

Reviewer #3: All comments have been addressed

Reviewer #4: All comments have been addressed

**********

2. Is the manuscript technically sound, and do the data support the conclusions?

The manuscript must describe a technically sound piece of scientific research with data that supports the conclusions. Experiments must have been conducted rigorously, with appropriate controls, replication, and sample sizes. The conclusions must be drawn appropriately based on the data presented.

Reviewer #1: Yes

Reviewer #3: Yes

Reviewer #4: Yes

**********

3. Has the statistical analysis been performed appropriately and rigorously?

Reviewer #1: N/A

Reviewer #3: Yes

Reviewer #4: Yes

**********

4. Have the authors made all data underlying the findings in their manuscript fully available?

The PLOS Data policy requires authors to make all data underlying the findings described in their manuscript fully available without restriction, with rare exception (please refer to the Data Availability Statement in the manuscript PDF file). The data should be provided as part of the manuscript or its supporting information, or deposited to a public repository. For example, in addition to summary statistics, the data points behind means, medians and variance measures should be available. If there are restrictions on publicly sharing data—e.g. participant privacy or use of data from a third party—those must be specified.

Reviewer #1: Yes

Reviewer #3: Yes

Reviewer #4: Yes

**********

5. Is the manuscript presented in an intelligible fashion and written in standard English?

PLOS ONE does not copyedit accepted manuscripts, so the language in submitted articles must be clear, correct, and unambiguous. Any typographical or grammatical errors should be corrected at revision, so please note any specific errors here.

Reviewer #1: Yes

Reviewer #3: Yes

Reviewer #4: Yes

**********

6. Review Comments to the Author

Please use the space provided to explain your answers to the questions above. You may also include additional comments for the author, including concerns about dual publication, research ethics, or publication ethics. (Please upload your review as an attachment if it exceeds 20,000 characters)

Reviewer #1: Paper is improved Paper is in publishable quality.

Please proofread the paper for possible further improvements.

Reviewer #3: The authors have satisfactorily addressed all of my comments.

One minor suggestion for the abstract, is to make it clearer that both RP and non-RP patients are from the same source:

The dataset held 200 color fundus images of Japanese RP patients and 121 color fundus images from non-

RP subjects from Keio University Hospital, and multiple images were available for some patients.

->

The dataset comprised of Japanese subjects from Keio University Hospital, which included 200 color fundus images from RP patients and 121 color fundus images from non-RP subjects. Multiple images were available for some patients.

I would also suggest giving the number of subjects rather than images since there are repeat images per per patient (if word limit allows).

Reviewer #4: One methodological issue that requires clarification is the handling of bilateral or multiple images from the same patient. The manuscript notes that some patients contributed more than one image, but it remains unclear whether data splitting was performed at the patient level. Because retinitis pigmentosa is generally bilateral, fellow-eye images from the same patient are not statistically independent and may show similar disease-specific fundus features. Therefore, image-level random splitting could introduce data leakage if images from the same patient were distributed across training and test sets. The authors should clarify this point and confirm that all images from a given patient were kept within the same split, or otherwise provide a patient-level sensitivity analysis.

**********

7. PLOS authors have the option to publish the peer review history of their article (what does this mean?). If published, this will include your full peer review and any attached files.

If you choose “no”, your identity will remain anonymous but your review may still be made public.

**Do you want your identity to be public for this peer review?**  For information about this choice, including consent withdrawal, please see our Privacy Policy.

Reviewer #1: No

Reviewer #3: No

Reviewer #4: No

**********

[NOTE: If reviewer comments were submitted as an attachment file, they will be attached to this email and accessible via the submission site. Please log into your account, locate the manuscript record, and check for the action link "View Attachments". If this link does not appear, there are no attachment files.]

To ensure your figures meet our technical requirements, please review our figure guidelines: https://journals.plos.org/plosone/s/figures

You may also use PLOS’s free figure tool, NAAS, to help you prepare publication quality figures: https://journals.plos.org/plosone/s/figures#loc-tools-for-figure-preparation.

NAAS will assess whether your figures meet our technical requirements by comparing each figure against our figure specifications.

# Author comment: Author response to Decision Letter 3

**DOI:** 10.1371/journal.pone.0354452.r006

21 Jun 2026

We have uploaded a detailed point-by-point response to the reviewers’ comments as a separate response document.

In brief, we revised the Abstract to clarify that both the retinitis pigmentosa and non-retinitis pigmentosa subjects were from Keio University Hospital and added the number of subjects as well as the number of images. We also clarified in the Methods section that all data splitting was performed at the patient level to prevent potential data leakage from bilateral or multiple images obtained from the same patient. In addition, we proofread the manuscript and made minor edits to improve clarity and readability.

In accordance with the journal requirements, we reviewed the reviewers’ comments and confirmed that no specific previously published works were recommended for citation in this round of review. We also reviewed the reference list to ensure that it is complete and accurate. No retracted articles were identified, and no changes were made to the reference list.

Attachment Submitted filename: Response to Reviewers June.docx

# Peer review: Decision Letter 3

**DOI:** 10.1371/journal.pone.0354452.r007

**Authors:** Vahid Mansouri

8 Jul 2026

Fundus Image Analysis of Retinitis Pigmentosa Using Artificial Intelligence

PONE-D-25-55224R3

Dear Dr. Kurihara,

We’re pleased to inform you that your manuscript has been judged scientifically suitable for publication and will be formally accepted for publication once it meets all outstanding technical requirements.

Within one week, you’ll receive an e-mail detailing the required amendments. When these have been addressed, you’ll receive a formal acceptance letter and your manuscript will be scheduled for publication.

An invoice will be generated when your article is formally accepted. Please note, if your institution has a publishing partnership with PLOS and your article meets the relevant criteria, all or part of your publication costs will be covered. Please make sure your user information is up-to-date by logging into Editorial Manager at Editorial Manager® and clicking the ‘Update My Information' link at the top of the page. For questions related to billing, please contact billing support.

If your institution or institutions have a press office, please notify them about your upcoming paper to help maximize its impact. If they’ll be preparing press materials, please inform our press team as soon as possible -- no later than 48 hours after receiving the formal acceptance. Your manuscript will remain under strict press embargo until 2 pm Eastern Time on the date of publication. For more information, please contact onepress@plos.org.

Kind regards,

Vahid Mansouri, M.D.

Academic Editor

PLOS One

Additional Editor Comments (optional):

Reviewers' comments:

Reviewer's Responses to Questions

**Comments to the Author**

1. If the authors have adequately addressed your comments raised in a previous round of review and you feel that this manuscript is now acceptable for publication, you may indicate that here to bypass the “Comments to the Author” section, enter your conflict of interest statement in the “Confidential to Editor” section, and submit your "Accept" recommendation.

Reviewer #3: All comments have been addressed

**********

2. Is the manuscript technically sound, and do the data support the conclusions?

The manuscript must describe a technically sound piece of scientific research with data that supports the conclusions. Experiments must have been conducted rigorously, with appropriate controls, replication, and sample sizes. The conclusions must be drawn appropriately based on the data presented.

Reviewer #3: Yes

**********

3. Has the statistical analysis been performed appropriately and rigorously?

Reviewer #3: Yes

**********

4. Have the authors made all data underlying the findings in their manuscript fully available?

The PLOS Data policy requires authors to make all data underlying the findings described in their manuscript fully available without restriction, with rare exception (please refer to the Data Availability Statement in the manuscript PDF file). The data should be provided as part of the manuscript or its supporting information, or deposited to a public repository. For example, in addition to summary statistics, the data points behind means, medians and variance measures should be available. If there are restrictions on publicly sharing data—e.g. participant privacy or use of data from a third party—those must be specified.

Reviewer #3: Yes

**********

5. Is the manuscript presented in an intelligible fashion and written in standard English?

PLOS ONE does not copyedit accepted manuscripts, so the language in submitted articles must be clear, correct, and unambiguous. Any typographical or grammatical errors should be corrected at revision, so please note any specific errors here.

Reviewer #3: Yes

**********

6. Review Comments to the Author

Please use the space provided to explain your answers to the questions above. You may also include additional comments for the author, including concerns about dual publication, research ethics, or publication ethics. (Please upload your review as an attachment if it exceeds 20,000 characters)

Reviewer #3: All my comments have been fully addressed.

One very minor correction:

Line 82: Eye2gene should be Eye2Gene

**********

7. PLOS authors have the option to publish the peer review history of their article (what does this mean?). If published, this will include your full peer review and any attached files.

If you choose “no”, your identity will remain anonymous but your review may still be made public.

**Do you want your identity to be public for this peer review?**  For information about this choice, including consent withdrawal, please see our Privacy Policy.

Reviewer #3: No

**********

# Editor report: Acceptance letter

**DOI:** 10.1371/journal.pone.0354452.r008

**Authors:** Vahid Mansouri

PONE-D-25-55224R3

PLOS One

Dear Dr. Kurihara,

I'm pleased to inform you that your manuscript has been deemed suitable for publication in PLOS One. Congratulations! Your manuscript is now being handed over to our production team.

At this stage, our production department will prepare your paper for publication. This includes ensuring the following:

* All references, tables, and figures are properly cited

* All relevant supporting information is included in the manuscript submission,

* There are no issues that prevent the paper from being properly typeset

You will receive further instructions from the production team, including instructions on how to review your proof when it is ready. Please keep in mind that we are working through a large volume of accepted articles, so please give us a few days to review your paper and let you know the next and final steps.

Lastly, if your institution or institutions have a press office, please let them know about your upcoming paper now to help maximize its impact. If they'll be preparing press materials, please inform our press team within the next 48 hours. Your manuscript will remain under strict press embargo until 2 pm Eastern Time on the date of publication. For more information, please contact onepress@plos.org.

You will receive an invoice from PLOS for your publication fee after your manuscript has reached the completed accept phase. If you receive an email requesting payment before acceptance or for any other service, this may be a phishing scheme. Learn how to identify phishing emails and protect your accounts at https://explore.plos.org/phishing.

If we can help with anything else, please email us at customercare@plos.org.

Thank you for submitting your work to PLOS ONE and supporting open access.

Kind regards,

PLOS ONE Editorial Office Staff

on behalf of

Dr. Vahid Mansouri

Academic Editor

PLOS One
